# Ultrasound evaluation of kidney and liver involvement in Bardet–Biedl syndrome

**DOI:** 10.1186/s13023-024-03400-w

**Published:** 2024-11-12

**Authors:** Metin Cetiner, Ilja Finkelberg, Felix Schiepek, Lars Pape, Raphael Hirtz, Anja K. Büscher

**Affiliations:** Children’s Hospital, Pediatrics II, Pediatric Nephrology, University of Duisburg-Essen, Essen, Germany

**Keywords:** Bardet–Biedl syndrome, Shear wave elastography, Attenuation imaging coefficient, Shear wave dispersion, Hepatic steatosis, Liver fibrosis, Renal disease

## Abstract

**Background:**

Bardet–Biedl syndrome (BBS) is a rare autosomal-recessive ciliopathy with pathogenic variants in 26 BBS genes. It affects multiple organs, including the kidney and liver, with varying degrees regarding extent and time of first manifestation. Structural renal anomalies are an early feature and end-stage kidney disease (ESKD) cumulates to 25% in adulthood. Early-onset hyperphagia-associated obesity is another major symptom and contributes to liver pathology, presenting as steatosis/fibrosis. Aim of this study is the evaluation of high-end ultrasound (US) technologies in BBS patients regarding their potential to discriminate liver and kidney tissue pathology at an early stage.

**Materials and methods:**

Patients with genetically proven BBS were recruited from the University Children’s Hospital of Essen and from BBS patient days hosted in Germany. Acute illness was an exclusion criterion. Clinical and laboratory data were extracted from patients’ digital records or medical letters. High-resolution ultrasound (US) imaging was utilized, including attenuation imaging (ATI), shear wave elastography (SWE) and dispersion (SWD) of liver tissue.

**Results:**

49 BBS patients (24/49 male; 1.1–51.0 years, mean 17.8 years) were included in the study. Mean body weight (SDS 2.13 ± 1.33) and BMI (SDS 2.64 ± 1.18) were increased. Structural kidney abnormalities (dysplasia, cysts) were present in 75% (36/48), and persistent fetal lobulation in 44% (21/48). Renal function was impaired in 27% (13/49) of whom 3 had ESKD (kidney transplantation (n = 2), hemodialysis (n = 1)). Elevation of liver enzymes was detected in 38% (16/42). In 51% (25/49) ATI of liver tissue was increased, indicating hepatic steatosis, and correlated with BMI SDS, liver size, and enzymes. SWE was elevated in 61% (30/49), suggesting hepatic fibrosis, and it correlated with BMI and GGT. Patients with pathogenic variants in *BBS10* showed a tendency towards higher ATI, reduced GFR, and higher BMI SDS.

**Conclusions:**

We detected kidney and liver abnormalities in a higher percentage of BBS patients than previously reported, indicating a high sensitivity and diagnostic yield of the evaluated high-end US applications. ATI detected liver pathology early (partially prior to liver enzymes) and revealed differences related to the affected genes. Evidence of tissue pathology at an early stage may improve diagnostics and the evaluation of therapeutic approaches.

**Supplementary Information:**

The online version contains supplementary material available at 10.1186/s13023-024-03400-w.

## Introduction

Bardet–Biedl syndrome (BBS) (OMIM#209,901; ORPHA: 110) is a rare ciliopathy with a prevalence of 1:160.000 and higher frequencies in isolated communities [1, 2]. At least 26 different BBS genes have been identified to date, with pathogenic variations in *BBS1* and *BBS10* accounting for 20–25% of patients in European countries. As ciliary-dependent pathways are essential in many cell types, multiple organs, including the brain, eye, skeleton, muscles, and the genitourinary system, are affected, however, to varying degrees and at different time points during childhood [2–8]. The cardinal feature at birth, with an overall frequency of > 50%, is renal anomalies, which might already present prenatally as hyperechogenic kidneys or later as structural abnormalities such as hypo-/dysplasia including parenchymal cysts together with impaired kidney function. The rate of end-stage kidney disease (ESKD) is reported to be less than 10% in preschool children but rises to 25% in adults, with a higher prevalence in females [9–12]. Another cardinal symptom is early-onset hyperphagia-associated obesity, which starts at preschool age and remains throughout life [13]. Obesity in BBS patients contributes to the development of metabolic disorders such as metabolic syndrome, type 2 diabetes [14–16], Metabolic Dysfunction-associated Steatotic Liver Disease (MASLD), and liver fibrosis (26–30%), all of which are related to an increased mortality at adult age [17, 18]. However, data to what extent liver involvement is secondary to obesity or due to dysfunctional cilia signaling, like in other related ciliopathies such as autosomal-recessive polycystic kidney disease or nephronophthisis, remains unclear [19–21].

A precise clinical description of these rare genetic disorders is of utmost importance for diagnostics, pathophysiological understanding, and the development of new therapeutic approaches. Ultrasound (US) technology represents an indispensable tool in diagnostics, support of interventions, and monitoring of therapeutic success. Over the last decades, US technology has developed continuously, particularly regarding image resolution and microvascular perfusion imaging [22]. Shear wave elastography (SWE), shear wave dispersion (SWD), and attenuation imaging coefficient (ATI) further improve liver assessment through the quantitative staging of liver fibrosis and steatosis as well as the detection of subtle changes in liver tissue at an early stage [23]. Thus, they provide diagnostic alternatives to MRI and CT scans with high applicability and acceptance rates in children [24] and may even replace invasive diagnostic procedures such as liver biopsy in the future.

The aim of our study was to perform a detailed evaluation of kidney and liver tissue in BBS patients with high-definition ultrasound technologies under consideration of genetic and clinical parameters.

## Materials and methods

### Patient recruitment and data collection

Between November 2020 and January 2023, 49 patients with genetically confirmed Bardet–Biedl syndrome were recruited. Thirty-seven of them attended the outpatient clinics of the Children`s Hospital of the University Duisburg-Essen. Twelve attended patient days organized by the Network for Early Onset of Cystic Kidney Diseases (NEOCYST) consortium [25]. Only patients with genetically confirmed Bardet–Biedl syndrome were included. The exclusion criteria were defined as follows: patients with any clinical signs of acute illness and patients with clinical but without genetically confirmed Bardet–Biedl syndrome. Non-fasting before the examination was not an exclusion criterion. Clinical and laboratory data were collected from digital patient records or medical letters. The local ethics committee approved the study. Written informed consent was obtained from all participants and/or parents/legal guardians, if appropriate. This study was conducted as part of the NEOCYST registry [25] in accordance with the Declaration of Helsinki on Biomedical Studies Involving Human Subjects.

### Standard ultrasound examination

Ultrasound examinations were performed using an Aplio i800 (Canon Medical Systems) with an i8CX1 matrix transducer (PVI-475BT, single curved, 1.8–6.2 MHz), enabling a detailed assessment of the liver and kidney parenchyma even in the presence of obesity. Two pediatricians specialized in pediatric ultrasonography (certified by the German Society of Ultrasound in Medicine and Biology, DEGUM) and long-standing experience in pediatric kidney and liver diseases performed upon availability and jointly reviewed all examinations. Examinations were conducted according to a defined setting: patients lay supine with both arms next to the body and were encouraged to breathe calmly (if possible regarding age). The duration of the examination, patient cooperation, and last food intake were documented. Standard ultrasound and Doppler examinations included abdominal wall thickness (measured from the cutis to the peritoneal layer adjacent to the liver capsule according to elastography measurements) and organ size and shape of the kidney, liver, spleen, and bladder. The kidney size was given as the volume (ml) derived from the measurements of length, width, and height. The liver size was measured in the sternal, midclavicular, and anterior axillary lines and determined by the mean of all three measurements. The dimension of the spleen was determined below the left costal margin. The results were given as the percentage of age- and height-related normal values [26]. Kidney ultrasound included an evaluation of echogenicity, corticomedullary differentiation, the presence of cysts, persistent fetal lobulation, urinary tract disorders, and velocity and flow profiles of the renal artery and renal vein. Pathological values for the Resistance Index (RI) and peak flow velocity of the renal artery were defined as follows: normal, decreased (> =  + 2SDS), or reduced (< = − 2SDS) [27, 28]. Liver ultrasound included evaluation of echogenicity, the shape of the lower liver margin, and parenchymal texture, including the presence of focal or diffuse lesions, dilation of the biliary tract, gallbladder abnormalities, and diameter, velocity, and flow profiles of hepatic arteries and veins.

### Shear wave elastography and dispersion of liver tissue

Shear wave speed was measured using an intercostal acoustic window (10 distinct measurements, liver segments V-VIII as recommended [29, 30]). Regions of interest (ROI, diameter 1cm) were placed at least 1 cm from the liver capsule and less than 6.3 cm from the skin. ROI placement avoided vessels and artifact areas. The mean and standard deviation values are given in kPa (elastography) and [m/s/kHz] (dispersion). The classification of SWE measurements exceeding the 97th percentile was based on published normal values in relation to abdominal wall thickness [23]. For abdominal wall thicknesses beyond the range covered by Cetiner et al. [23], SWE values were classified as pathological (> 97th percentile) when exceeding 6 kPa, consistent with published normal values for adults [31]. SWD values were classified based on published normal values in relation to BMI SDS levels [23]. SWD levels in patients with a BMI SDS >  + 2 SDS were classified as exceeding the 97th percentile if the corresponding SWD value was above 14.6 [(m/s)/kHz] (> + 2SD) in adults according to published data [31].

### Attenuation imaging

Five distinct liver attenuation-imaging measurements were performed for every patient (trapezoidal ROI avoiding areas too close to the liver capsule, larger vessels, and artifacts). A quality measure of the liver ATI coefficient correlating the attenuation with the depth (goodness of fit—R^2^) was provided. The R^2^ values were categorized into poor (R^2^ < 0.80), good (0.80 ≤ R^2^ < 0.90), and excellent (R^2^ > 0.90), and only excellent values with R^2^ > 0.90 were accepted. The mean and standard deviation of the attenuation coefficient in [dB/cm/MHz] are reported. In children and adolescents, the classification of an ATI measurement exceeding the 97th percentile was based on age-dependent normal values [23]. For adults with BBS, values exceeding 0.63 [dB/cm/Mhz] were classified above the 97th percentile [23, 32].

## Statistical analyses

### Methods

SPSS 29.0 (Armonk, NY: IBM Corp.) and R (version 4.2.1, R Core team, 2022), as well as the R-packages FWDselect [version 2.1.0 [33]] and lmtest [version 0.9–40 [34]] were used for data handling and analysis.

Post-hoc power analyses were performed with GPower (3.1, HHU Düsseldorf [35]), assuming α = 0.05 and β = 0.80, and the results were interpreted in terms of Cohen’s *d* (small 0.21 ≤ *d* ≤ 0.49, medium 0.50 ≤ *d* ≤ 0.79, large ≥ 0.8 [36]).

Analyses were either FDR-corrected at *p* < 0.05 (two-tailed) for multiple comparisons or considered exploratory, as described in the respective section below.

### Descriptive statistics and correlation analysis

Prior to analysis, data pertaining to ATI, SWE, and SWD were winsorized. This procedure refers to replacing outliers with predefined values. Following recent recommendations [37], outliers were designated by values exceeding ± 2.5 the median absolute difference (MAD) and replaced by values corresponding to ± 2.5 times the MAD concerning the variable of interest.

Continuous variables pertaining to demographic characteristics were compared between the BBS and the normative sample by either t-tests or median-tests (details on testing the statistical assumptions of the employed tests are provided in the Supplementary Material). The distribution of categorical variables in both samples was compared by z-tests.

Bivariate correlation analyses were conducted using Kendall’s *τ*. This measure allows for considering variables of any scale, including dichotomous nominal variables, within a robust statistical framework [38]. The comparison of demographic characteristics between samples and the analysis of bivariate correlations was deemed exploratory at *p* < 0.05.

### Multiple regression

Considering a large number of potential covariates with complete information, most of which have been considered by previous studies (e.g., age [years], sex, height-SDS, weight-SDS, BMI-SDS, abdominal wall thickness [mm], fasting duration [hours], cooperation [calm vs. restless; dummy-coded], liver echogenicity, liver lower edge [dichotomized and dummy-coded: concave shape; pointed shape, rounded shape], liver size standardized to height-related mean value in %, spleen size standardized to height-related mean value in %), a two-step procedure as implemented in the FWRselect R-package was used to identify the most appropriate subset of covariates (independent variables) concerning the analysis of ATI, SWE, and SWD levels (dependent variables) in BBS patients by multiple linear regression [33]. As previously described [23], a greedy forward selection algorithm was employed in the first step, changing one variable at a time until no further improvement in model fit assessed by the Akaike information criterion (AIC) was attained. Second, a bootstrap-based procedure evaluating the number of significant covariates as a trade-off between model size and model fit was performed at a significance level of *p* < 0.05. All results were FDR-corrected for multiple comparisons. Assumptions concerning the resulting multiple regression models were assessed as detailed in the Supplementary Material.

### Group comparisons

Analyses of covariance (ANCOVA) were performed to compare ATI, SWE, and SWD levels within the group of BBS patients by their genotype and between the total sample of BBS patients and the norming sample. Regarding the former comparison, these analyses accounted for covariates identified by the previous step of analysis detailed above. This also applied to the comparison of BBS patients and the norming sample. However, these analyses also considered covariates related to ATI, SWE, and SWD levels established in the norming sample [23] as well as demographic characteristics significantly different between both samples. All results were FDR-corrected for multiple comparisons [39].

## Results

### Patient characteristics

A total of 49 BBS patients (aged 1.1–51.0 years, mean 17.8 years, median 16.8 years) were included in this prospective study. The sex distribution was near-balanced with 49% (24/49) male patients. The body length was normally distributed; the mean weight SDS (2.13) and mean BMI SDS (2.64) were well above the 97th percentile with higher values in children compared to adults (mean BMI z-score 2.77 vs. 2.50; Table 1).

**Table 1:** Demographics and selected blood laboratory data in BBS patients

	All (n = 49)	Children (n = 27)	Adults (n = 22)
Gender M	49.0% (n = 24)	48.1% (n = 13)	50.0% (n = 11)
F	51.0% (n = 25)	51.9% (n = 14)	50.0% (n = 11)
Age (years)	17.8 ± 11.4 (16.8; 1.1–51)	9.7 ± 5.1 (10.8; 1.1–17.6)	27.6 ± 9.0 (24.5; 18–51)
Height (cm)	151.7 ± 26.6 (161; 81–191)	138.2 ± 28.3 (148; 81–178.9)	168.4 ± 9.2 (169.5; 150.6–191)
Weight (kg)	77.8 ± 35.8 (85; 16.0–139.7)	62.8 ± 35.8 (62.5; 16.0–139.7)	96.2 ± 25.8 (93.8; 44.7–139.7)
BMI (kg/m^2^)	31.5 ± 9.2 (30.8, 18.6–55.2)	29.5 ± 8.9 (26.0, 18.9–55.2)	33.9 ± 9.0 (31.6, 18.6–50.7)
Height SDS	0.02 ± 1.29 (-0,05; -3.01–3.04)	0.25 ± 1.38 (0.16; -3.01–3.04)	-0.23 ± 1.13 (-0,26; -2.20–2.00)
Weight SDS	2.13 ± 1.33 (2.34; -2.09–4.91)	2.62 ± 1.18 (2.79; 0.50–4.91)	1.57 ± 1.24 (1.84; -2.09–2.90)
BMI SDS	2.64 ± 1.18 (2.62; -1.22–4.71)	2.77 ± 0.91 (2.85; 0.75–4.43)	2.50 ± 1.45 (2.35; -1.22–4.71)
GFR reduced	27% (13/49)	26% (7/27)	27% (6/27)
Kreatinin (umol/l)	92 ± 89 (66; 25–494)	67 ± 42 (57; 25–249)	127 ± 120 (80; 56–494)
GOT/AST (U/l)	38 ± 31 (28; 16–210)	45 ± 38 (41; 16–210)	28 ± 12 (25; 16–68)
GPT/ALT (U/l)	41 ± 35 (29; 9–195)	43 ± 37 (33; 15–195)	37 ± 30 (26; 9–139)
GGT (U/l)	47 ± 73 (20; 8–325)	48 ± 83 (19; 8–325)	46 ± 59 (22; 12–240)
AP (U/l)	186 ± 115 (144; 25–427)	250 ± 106 (255; 25–427)	98 ± 50 (94; 27–222)

M—male / F—female / n—sample size

GFR—glomerular filtration rate

GOT—glutamic oxaloacetic transaminase/AST—aspartate aminotransferase

GPT—glutamic pyruvic transaminase/ALT—alanine aminotransferase

GGT—gamma-glutamyltransferase

AP—alkaline phosphatase

**Fig. 1:**
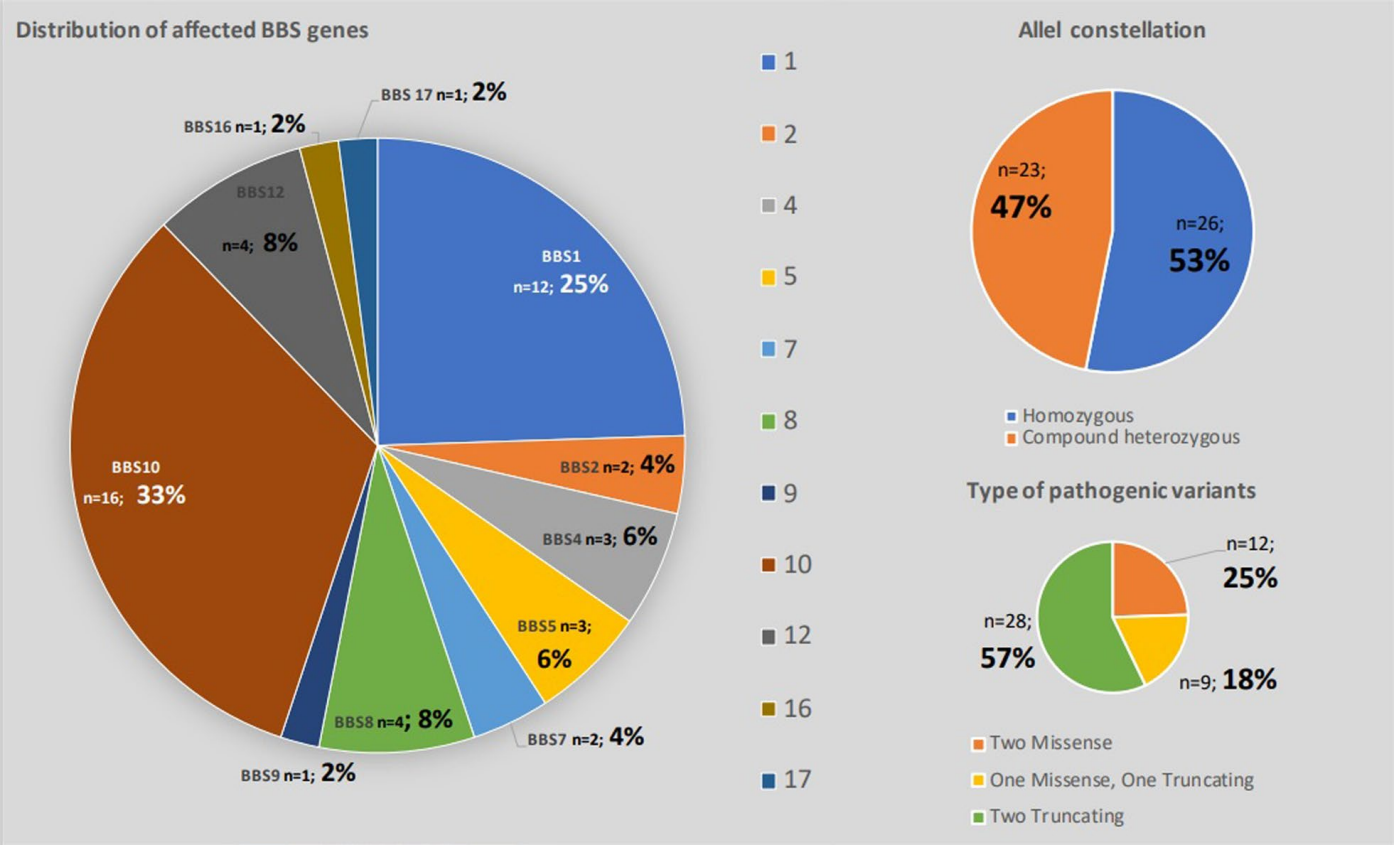
Genetic findings in the BBS study cohort

Pathogenic variants in BBS genes 10 (16/49–33%) and 1 (12/49–25%) were the most common variants and accounted for 58% of the study cohort. The hot spot mutations in the *BBS1* gene (BBS: *c.1169T* > *G*; p.Met390Arg) and the *BBS10* gene (*c.271dupT*; p.Cys91Leufs*5) were prevalent in 10 patients each (*BBS1*: n = 7 homozygous, n = 3 compound heterozygous, 71% of all *BBS1* patients; *BBS10*: n = 4 homozygous, n = 6 compound heterozygous, 44% of all *BBS10* patients). Our cohort included patients with pathogenic variants in nine further BBS genes (Fig. 1). Homozygous variants were more frequent (26/49–53%) than compound heterozygous variants. Two truncating variants (28/49–57%) were more common than two missense variants or a combination of missense and truncating variants.

Impairment of renal function was present in 27% (13/49). Three patients (6%) developed ESKD at ages 6, 10, and 46 years. Two adult patients were under dialysis treatment (hemodialysis (n = 1) and peritoneal dialysis after failure of a kidney transplant due to chronic humoral rejection (n = 1)), and one 17-year-old patient had received a kidney transplant (eGFR 63 ml/min/1.73m^2^, homozygous truncating variant in *BBS16*). Liver enzymes (GOT, GPT, GGT) were elevated in 38% (16/42; at least one parameter). The most common long-term medication was vitamin D (53%, 26/49); others included oral iron supplementation (12%, 6/49) and l-thyroxine (10%, 5/49) replacement therapy. In individual cases, medication was taken for sleep disorders, behavioral problems, seizures, high blood pressure, and steroid-free immunosuppression (cyclosporine, tacrolimus, mycophenolate mofetil) after kidney transplantation.

### Technical characteristics of ultrasound examination

Ultrasound examination was overall (86%, 42/49) well tolerated (“good” cooperation) with lower rates in children compared to adults (78% [21/27] vs. 95% [21/22]). The mean duration of ultrasound examination of the liver (basic ultrasound + SWE, SWD, and ATI assessment) was 7.5 ± 2.6 min (median 7 min, range 4 to 15 min) and comparable between children and adults (7.6 vs. 7.4 min). Last food intake was more than 2 h ago in 71% (35/49). The mean abdominal wall thickness was 22.5 ± 7.5 mm (median 22, range 9 to 43; Table 2).

**Table 2:** Ultrasound findings including liver, spleen, and kidney in patients with BBS

	All (n = 49)	Children (n = 27)	Adults (n = 22)
Cooperation US exam			
Calm	85.7% (n = 42)	77.8% (n = 21)	95.5% (n = 21)
Restless	14.3% (n = 7)	22.2% (n = 6)	4.5% (n = 1)
Fasting since			
< 1 h	20.4% (n = 10)	22.2% (n = 6)	18.2% (n = 4)
< 2 h	8.2% (n = 4)	11.1% (n = 3)	4.5% (n = 1)
< 3 h	10.2% (n = 5)	11.1% (n = 3)	9.1% (n = 2)
< 4 h	34.7% (n = 17)	33.3% (n = 9)	36.4% (n = 8)
> 4 h	26.5% (n = 13)	22.2% (n = 6)	31.8% (n = 7)
LIVER + SPLEEN			
Duration US liver exam (min)	7.5 ± 2.6 (7; 4–15)	± 2.3 (7; 4–13)	7.4 ± 2.9 (6; 5–15)
Liver Size (% relative to the mean)	114 ± 18 (111; 86–158)	116 ± 20 (110; 86–158)	112 ± 16 (112; 86–152)
Spleen Size (% relative to the mean)	108 ± 12 (107; 80–140)	106 ± 13 (106; 80–134)	109 ± 12 (108; 94–140)
Abdominal wall thickness (mm)	22.5 ± 7.5 (22;9–43)	21.3 ± 5.8 (23; 9–31)	24.1 ± 8.9 (20.5; 9–43)
Liver lower edge			
Concave shape	75.5% (n = 37)	74.1% (n = 20)	77.3% (n = 17)
Pointed shape	14.3% (n = 7)	14.8% (n = 4)	13.6% (n = 3)
Rounded shape	10.2% (n = 5)	11.1% (n = 3)	9.1% (n = 2)
Liver echogenicity			
Normal	65.3% (n = 32)	66.7% (n = 18)	63.6% (n = 14)
Increased	34.7% (n = 17)	33.3% (n = 9)	36.4% (n = 8)
Decreased	0% (n = 0)	0% (n = 0)	0% (n = 0)
Gallbladder			
Empty	18.4% (n = 9)	25.9% (n = 7)	9.1% (n = 2)
Medium full Full	22.4% (n = 11) 55.1% (n = 27)	18.5% (n = 5) 55.6% (n = 15)	27.3% (n = 6) 54.5% (n = 12)
Removed	4.1% (n = 2)	0% (n = 0)	9.1% (n = 2)
A. hepatica systolic flow velocity (cm/s)	60.6 ± 36.4 (51; 30–189)	64.6 ± 38.1 (52; 30–182)	57.3 ± 34.6 (47.5; 31–189)
V. porta flow velocity (cm/s)	28.6 ± 8.5 (26.5; 17–51)	31.2 ± 8.8 (30; 18–51)	26.3 ± 7.6 (26; 17–50)
V. hepatica flow velocity (cm/s)	40.3 ± 16.3 (41; 18–85)	41.2 ± 16.9 (41; 18–78)	39.6 ± 15.6 (38; 20–85)
V. hepatica flow profile			
Triphasic flow pattern	49.0% (n = 24)	37.0% (n = 10)	63.6% (n = 14)
Limited triphasic flow pattern	16.3% (n = 8)	18.5% (n = 5)	13.6% (n = 3)
Biphasic flow pattern	18.4% (n = 9)	18.5% (n = 5)	18.2% (n = 4)
Monophasic flow pattern	0% (n = 0)	0% (n = 0)	0% (n = 0)
Not measured	16.3% (n = 8)	25.9% (n = 7)	4.5% (n = 1)
KIDNEY			
Total kidney volume (% relative to the mean)	115 ± 42 (105; 49–224)	102 ± 34 (93; 49–182)	131 ± 45 (137; 57–224)
Right Kidney volume (% relative to the mean)	115 ± 48 (105; 43–273)	98 ± 32 (94; 48–190)	136 ± 56 (126; 43–273)
Left Kidney volume (% relative to the mean)	115 ± 48 (102; 22–214)	106 ± 41 (101; 35–214)	126 ± 53 (116; 22–214)
Volume (right-sided; ml)	109.3 ± 58.2 (101.5; 24–293)	86.3 ± 42.7 (84; 24–170)	138.9 ± 62.0 (125; 46–293)
Volume (left-sided; ml)	111.1 ± 60.2 (102.3; 17–230)	87.7 ± 50.9 (86; 17–230)	141,1 ± 57.9 (147; 24–230)
Echogenicity (right-sided)			
Normal	52.1% (n = 25)	40.7% (n = 11)	66.7% (n = 14)
Increased	47.9% (n = 23)	59.3% (n = 16)	33.3% (n = 7)
Echogenicity (left-sided)			
Normal	47.9% (n = 23)	33.3% (n = 9)	66.7% (n = 14)
Increased	52.1% (n = 25)	66.7% (n = 18)	33.3% (n = 7)
Corticomedullary-differentiation (right-sided)			
Normal	37.5% (n = 18)	25.9% (n = 7)	52.4% (n = 11)
Reduced	29.2% (n = 14)	29.6% (n = 8)	28.6% (n = 6)
Non-existent	33.3% (n = 16)	44.4% (n = 12)	19.0% (n = 4)
Corticomedullary-differentiation (left-sided)			
Normal	39.6% (n = 19)	25.9% (n = 7)	57.1% (n = 12)
Reduced	31.2% (n = 15)	37.0% (n = 10)	23.8% (n = 5)
Non-existent	29.2% (n = 14)	37.0% (n = 10)	19.0% (n = 4)
Persistent fetal lobulation (right-sided)			
No	56.3% (n = 27)	63.0% (n = 17)	47.6% (n = 10)
Yes	43.7% (n = 21)	37.0% (n = 10)	52.4% (n = 11)
Persistent fetal lobulation (left-sided)			
No	60.4% (n = 29)	66.7% (n = 18)	52.4% (n = 11)
Yes	39.6% (n = 19)	33.3% (n = 9)	47.6% (n = 10)
Urinary tract disorder (right-sided)			
No	91.7% (n = 44)	88.9% (n = 24)	95.2% (n = 20)
Yes	8.3% (n = 4)	11.1% (n = 3)	4.8% (n = 1)
Urinary tract disorder (left-sided)			
No	89.6% (n = 43)	88.9% (n = 24)	90.5% (n = 19)
Yes	10.4% (n = 5)	11.1% (n = 3)	9.5% (n = 2)
A. renalis systolic flow velocity (m/s) (right-sided)	75.2 ± 21.3 (73; 43–125)	76.5 ± 24.5 (76; 43–125)	72.9 ± 13.6 (73; 48–94)
A. renalis systolic flow velocity (m/s) (left-sided)	67.2 ± 21.0 (61; 38–132)	74.2 ± 22.0 (73; 43–132)	55.2 ± 11.7 (56; 38–72)
A. renalis resistance index (RI) (right-sided)	0.68 ± 0.09 (0.68; 0.36–0.82)	0.69 ± 0.10 (0.70; 0.36–0.82)	0.65 ± 0.07 (0.66; 0.47–0.73)
A. renalis resistance index (RI) (left-sided)	0.70 ± 0.06 (0.71; 0.50–0.80)	0.71 ± 0.06 (0.71; 0.50–0.80)	0.69 ± 0.05 (0.70; 0.58–0.75)
Bladdervolume unusual full			
No	83.3% (n = 40)	81.5% (n = 22)	85.7% (n = 18)
Yes	16.7% (n = 8)	18.5% (n = 5)	14.3% (n = 3)
2 RT, 2 NC 1 (adults) 1 pelvic kidney left-sided (child)			

hr/hrs—hour/hours; RT—renal transplantation; NC—nephrocalcinosis

### Kidney ultrasound

In pediatric patients, the overall mean weight-adjusted total kidney volume was normal with a wide distribution (Table 2). The total kidney volume in adults with absolutely higher body weights was correspondingly higher. The mean resistance index (RI) was within the normal range, but 55% (17/31) exhibited an increased RI value. This applied to all patients with an impaired GFR. Mean renal artery peak flow velocity was within the normal range, with reduced velocity observed in 10% (28/31).

**Fig. 2:**
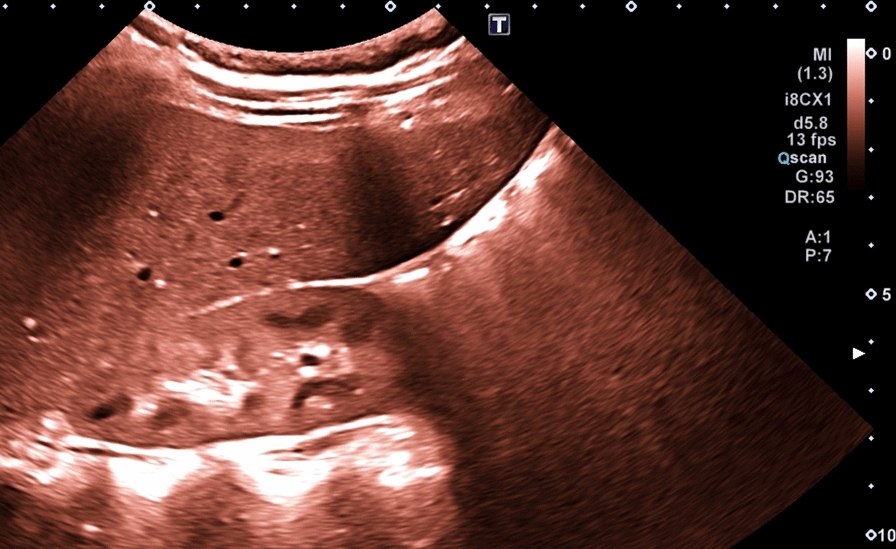
Increased kidney echogenicity and preserved corticomedullary differentiation without persistent fetal lobulation in an 11-year old BBS female patient (patient ID37; pathogenic homozygous variant in BBS 17 gene; c.778–3 C > T) and video representation in Supplement Fig. 2

**Fig. 3:**
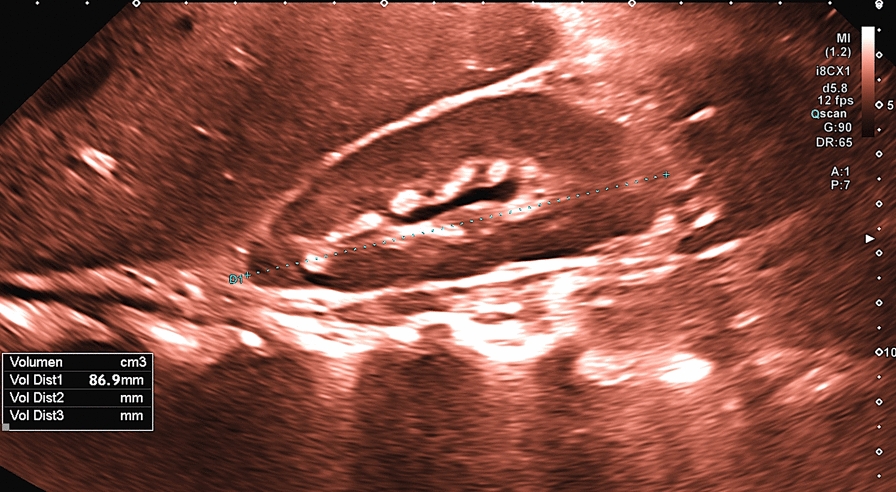
Increased kidney echogenicity and non-existent corticomedullary differentiation in a female 9-year old BBS patient (patient ID26; pathogenic compound heterozygous variant in BBS 5 gene; c.54dupC p. (ala19Argfs*14) + deletion Exon 10–12)

**Fig. 4:**
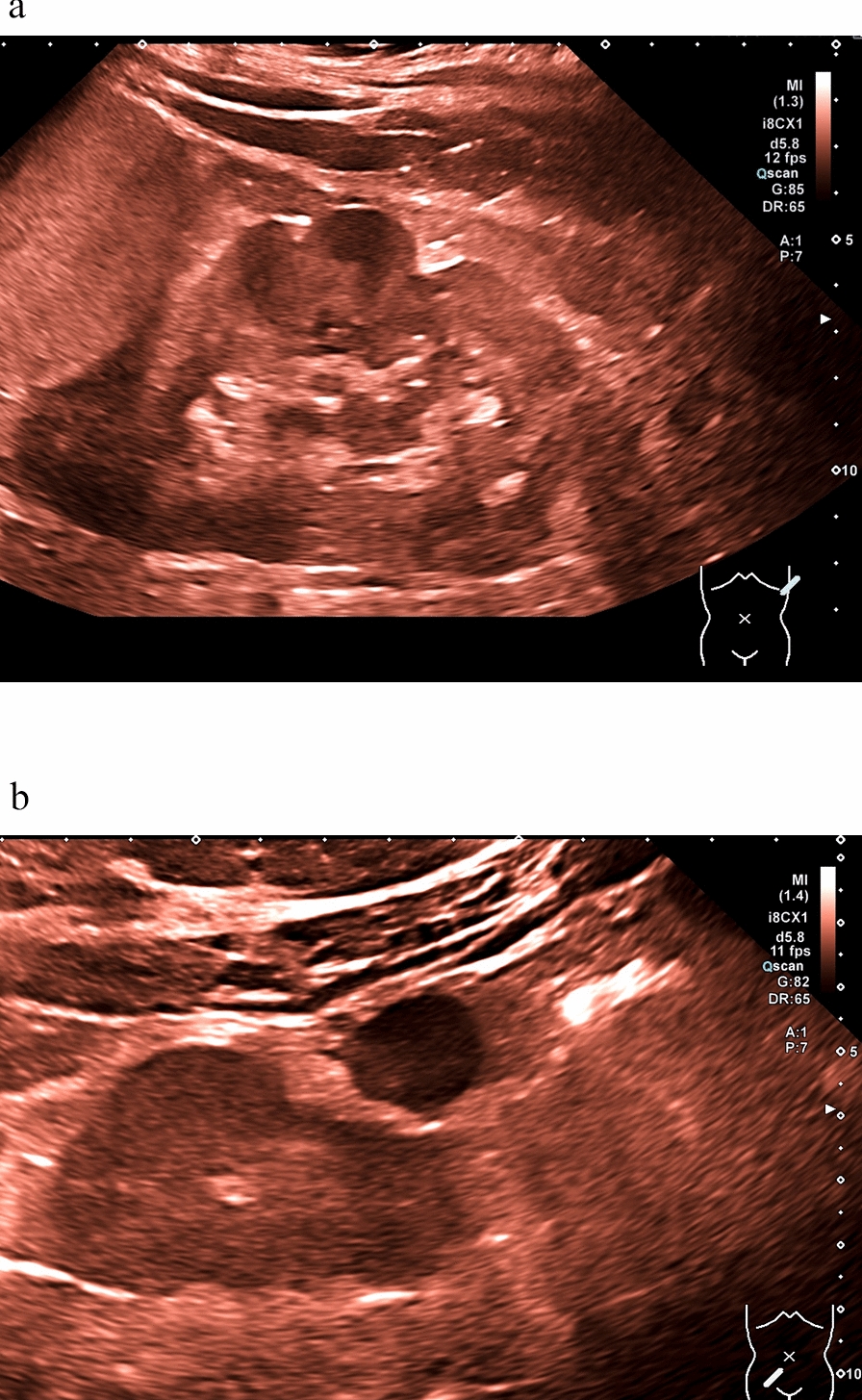
**a** Left kidney with significant persistent fetal lobulation with increased kidney echogenicity and almost complete diminished corticomedullary differentiation in a 10-year old female BBS patient (patient ID5; pathogenic homozygous variant in BBS 8 gene; deletion Exon 9) and video representation in Supplement Fig. 3; **b** Hypoplastic right pelvic kidney with non-existent corticomedullary differentiation and incidental findings of right-sided ovarian cyst (same patient as in Fig. 4a)

Overall, kidney structure abnormalities were present in 75% of patients (36/48). Increased renal echogenicity and decreased or increased corticomedullary differentiation, as indicators for renal abnormalities, were observed in 52% (25/48) and 63% (30/48) of the cohort, respectively (Figs. 2, 3, and 4). Renal cysts were prevalent in 21% (10/48).

Persistent fetal lobulation appeared in 44% (21/48). Urinary tract disorders were less common and presented exclusively as mild urinary tract dilatations (10.4%, 5/48). A bladder volume beyond the age-specific range (above 400 ml in adults, [years of age + 2] × 30 ml in children [40]) was detected in 20% of patients. Two cases presented with nephrocalcinosis grade 1, and one child had a left-sided pelvic kidney. Native kidneys were not detectable in one patient after kidney transplantation (Table 2).

### Liver ultrasound

The sizes of the liver and spleen were age- and height-adjusted and slightly increased (mean 114% and 108%, respectively). Organ perfusion was measured in cm/s and showed no anomalies (Table 2). Increased liver echogenicity was present in 17/49 (35%). A minority of patients (10%, 5/49) exhibited a rounded lower liver margin as one major US sign for parenchymal abnormalities. In this subgroup, the mean values for BMI SDS (3.72), liver size (140%), SWE (6.8 kPa), and ATI (0.73 dB/cm/Mhz) were descriptively elevated compared to the present study cohort. According to the high percentage of patients with a fasting period > 2 h, the filling state of the gallbladder was moderate (23%, 11/47) or high (57%, 27/47) in the majority of patients.

### SWE, SWD, and ATI

**Fig. 5:**
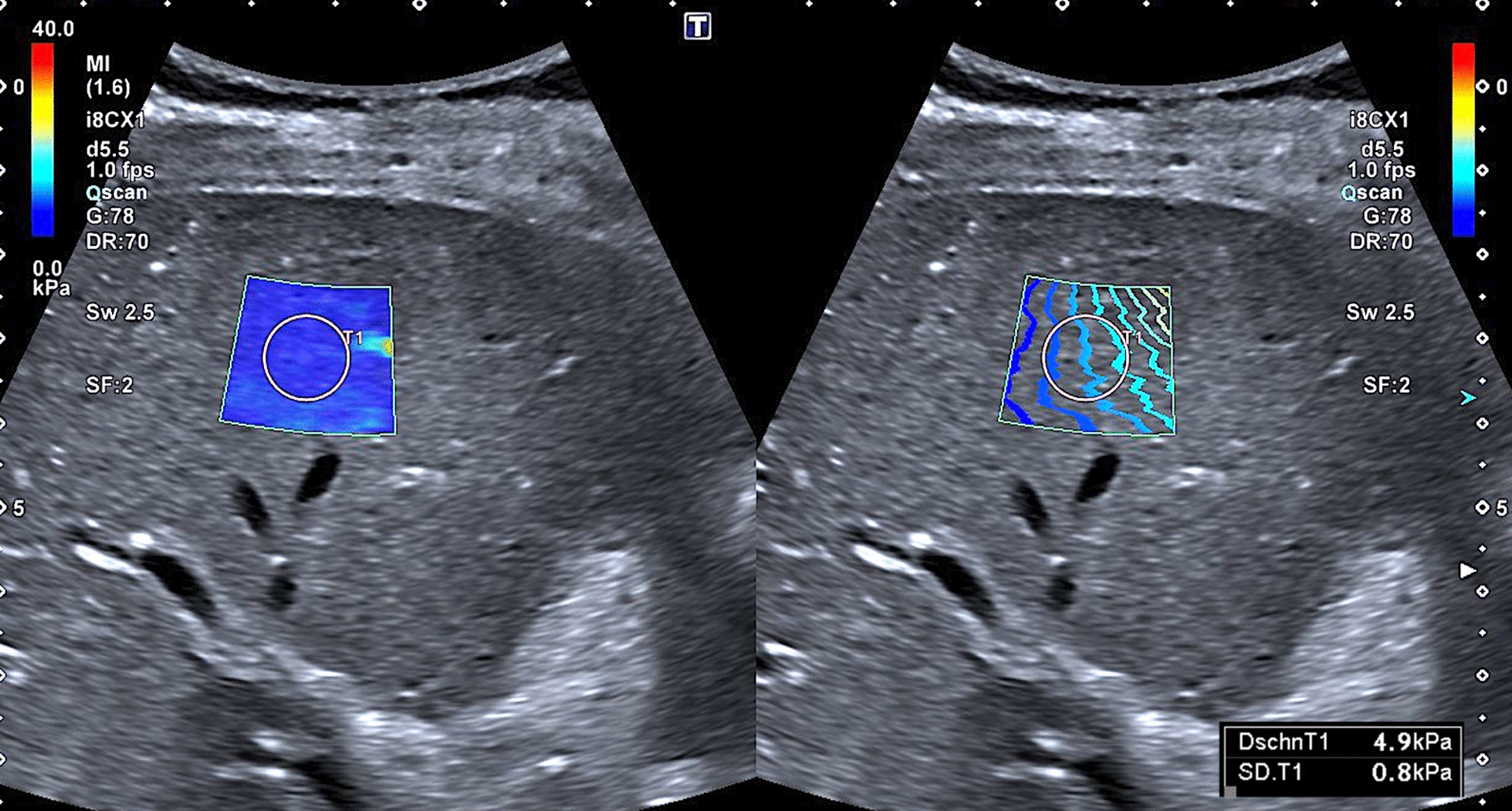
Normal liver parenchymal structure in in a 18-year old female BBS patient (patient ID34; pathogenic compound heterozygous variant in BBS 2 gene; c.823C > T; p.Arg275*) + c.1986dupT (p.Asn663*)

**Fig. 6:**
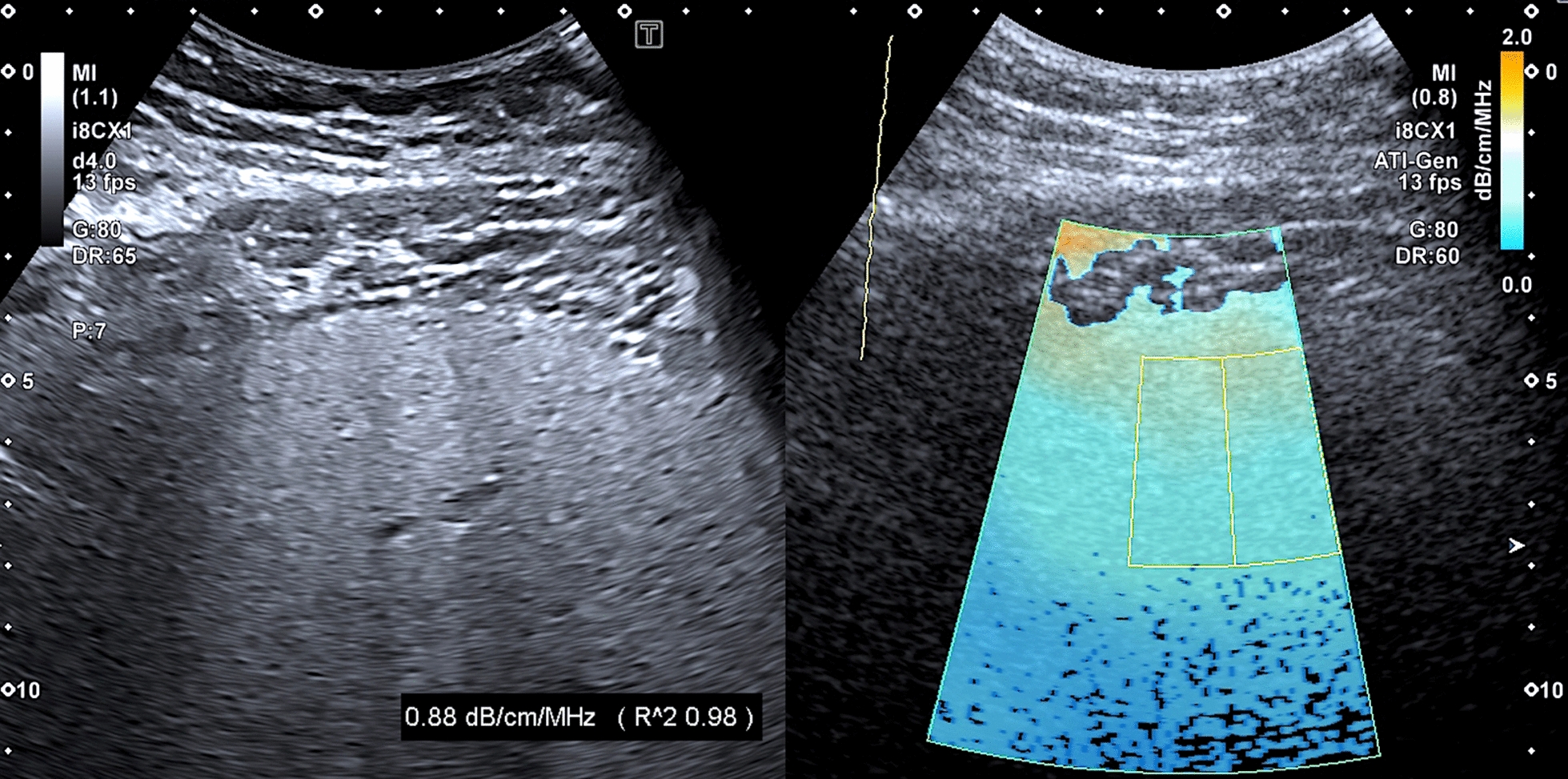
High-grade steatosis in in a 19-year old male BBS patient (patient ID 42; pathogenic homozygous variant in BBS 10 gene; c.1269_1273del (p.Gln423fsX))

**Fig. 7:**
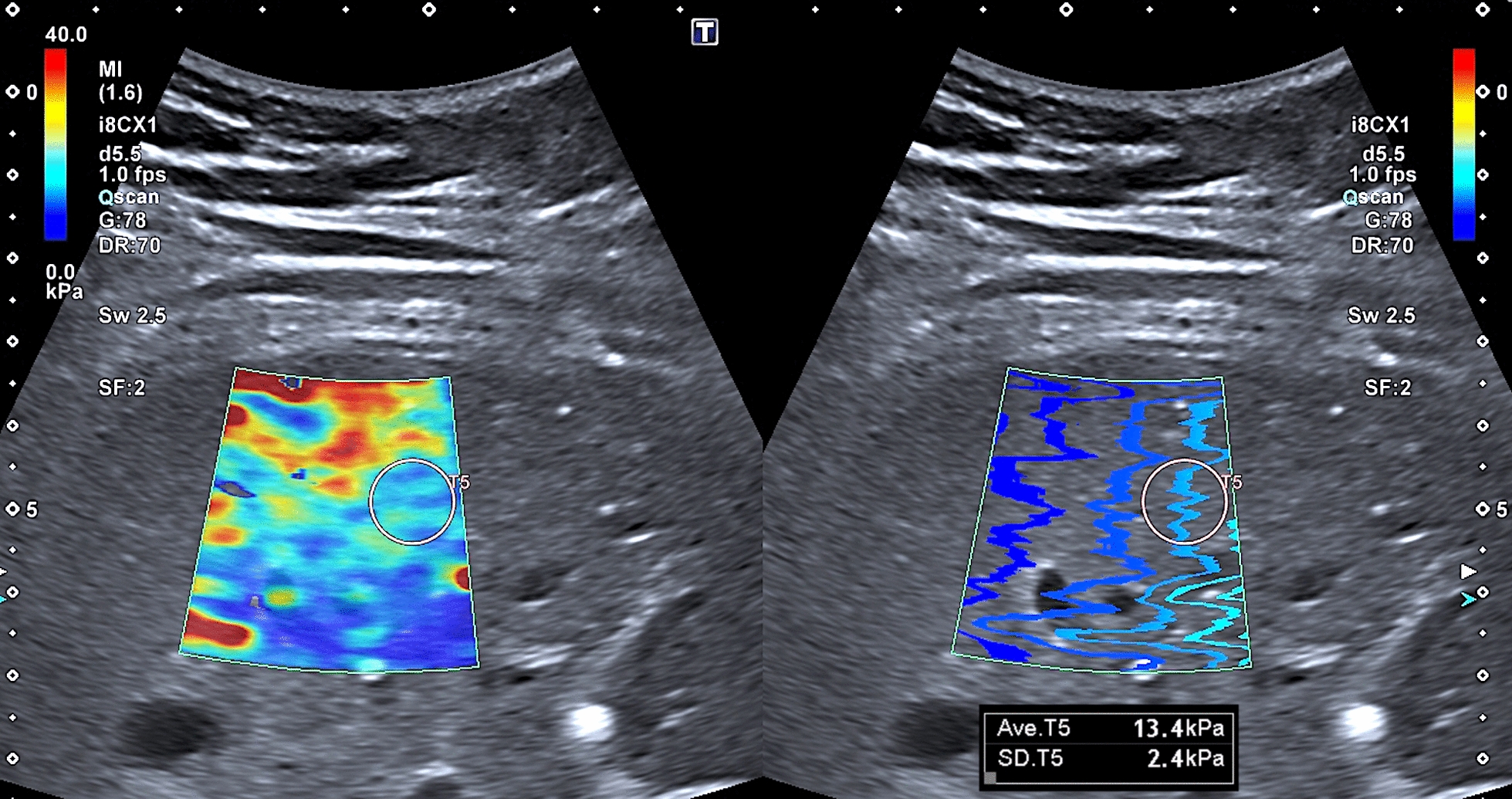
High-grade liver fibrosis in a 31-year old female BBS patient (patient ID49; pathogenic homozygous variant in BBS 1 gene; c.1169 T > G p.(Met390Arg)

**Fig. 8:**
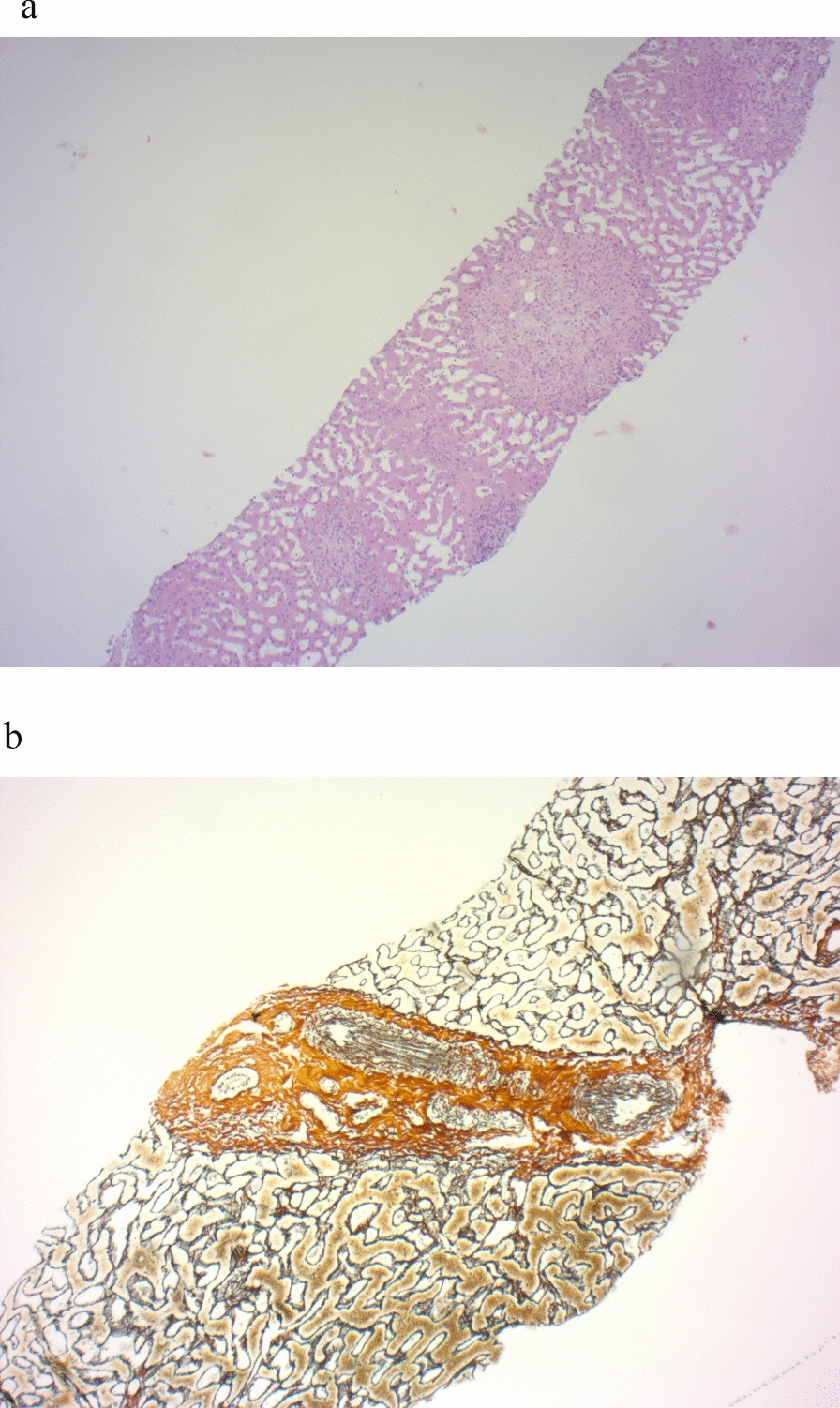
**a** Same patient as in Fig. 7: Overview in the HE staining. Liver parenchyma with epitheloid cellular granulomas (objective 4× corresponding to 40× total magnification) ectatic sinusoids (objective 10× corresponding to 100× total magnification) as signs of severe chronic portal moderate active inflammation of the liver tissue with florid (non-purulent destructive) bile duct lesions, partial ductopenia of local bile ducts and septate fibrosis without complete cirrhotic remodeling (stage 2/4 according to Desmet). No fatty degeneration. No siderosis. **b** Same patient as in Fig. 7: Gomori staining to visualize the hepatic fibrous tissue. Fibrosis in a portal field but overall no cirrhotic remodeling (objective 10× corresponding to 100× total magnification)

The results of measurements are given in Table 3. Overall, the mean ATI values were above the normal range, with descriptively slightly higher values in children than in adults. Among BBS patients, 51% (25/49) had ATI values above the 97th percentile and an additional 16% (8/49) between the 90th and 97th percentiles. The majority of BBS patients demonstrated SWE values above the 97th percentile (61%, 30/49), with higher values in adults. SWE values equal to or above 7.0 kPa, indicating hepatic fibrosis [41], were measured in 31% (15/49) of the cohort (Figs. 5, 6, 7, and 8). Regarding SWE, ROIs were placed at a mean of 4.3 ± 0.84 cm below the skin (median 4.3 cm; range 2.8 to 6.3 cm, recommended < 5 cm). The mean SWD level was in the normal range, corresponding to the 50th percentile in children (13.0 [(m/s)/kHz]) and the 75th percentile in adults (13.7 [(m/s)/kHz]) when considering BMI. However, 23% of the cohort (9/39) demonstrated SWD levels above the 97th percentile indicating elevated liver viscosity. This mainly applied to male BBS patients (89%, 8/9), patients with a pathogenic variant in the *BBS10* gene (56%, 5/9), and those with elevated ATI (0.71 dB/cm/Mhz) and SWE levels (6.8 kPa).

**Table 3:** Ultrasound findings of liver parenchyma using ATI, SWE, and SWD in patients with BBS

	All (n = 49)	Children (n = 27)	Adults (n = 22)
ATI Mean (dB/cm/MHZ)	0.68 ± 0.11 (0.66; 0.46–0.91)	0.70 ± 0.09 (0.68; 0.55–0.91)	0.65 ± 0.12 (0.63; 0.46–0,90)
SWE Mean (m/s)	1.44 ± 0.16 (1.39; 1.15–1.92)	1.40 ± 0.14 (1.34; 1.15–1.80)	1.50 ± 0.17 (1.50; 1.28–1.92)
SWE Mean (kPa)	6.3 ± 1.5 (5.6; 3.8–11.2)	5.8 ± 1.3 (5.3; 3.8–9.7)	6.8 ± 1.7 (6.7; 4.7–11.2)
SWD Mean [(m/s)/kHz]	13.4 ± 2.1 [13.2; 9.4–18.3)	13.0 ± 2.1 (12.9; 9.4–17.3)	13.7 ± 2.1 (13.4; 10.7–18.3)

### Correlation analysis

A minor proportion of SWE (2%, 1/49) and SWD (2%, 1/49) measurements was winsorized prior to further analyses. This is well below a recommended threshold of 5% [35].

Exploratory bivariate analyses revealed no relationship between ATI, SWE, and SWD levels (Table 5). Concerning auxological parameters, ATI levels were positively correlated with BMI-SDS (*r*(47) = 0.31; *p* = 0.002). This also applied to abdominal wall thickness (*r*(47) = 0.32; *p* = 0.001) and confirmed the former finding. Liver size (%) and echogenicity were positively related to ATI measurements, consistent with a positive relationship with GOT (*r*(39) = 0.22; *p* = 0.05), GPT (*r*(40) = 0.33; *p* = 0.003) and GGT levels (*r*(39) = 0.29; *p* = 0.01) in a subsample of BBS patients. Also, spleen (*r*(43) = 0.27; *p* = 0.01) and kidney size (*r*(45) = 0.25; *p* = 0.02) were positively correlated with ATI levels in subsample analyses.

There was a similar relationship of SWE measurements with BMI-SDS (*r*(47) = 0.25; *p* = 0.012) and abdominal wall thickness (*r*(47) = 0.35; *p* = 0.001) as observed for ATI levels. However, apart from GGT levels (*r*(39) = 0.22; *p* = 0.045), SWE measurements were neither related to liver morphology, liver enzymes, or other organ status.

There was a statistical trend (0.05 < *p* < 0.10) towards higher SWD levels in males than in females (*r*(37) = − 0.23; *p* = 0.09). Moreover, SWD levels were lower in patients with pathological kidney findings (*r*(34) = − 0.37; *p* = 0.006; 0 = GFR within age range, kidney size and normal echogenicity; 1 = GFR within age range, increased echogenicity [at least one kidney] and/or reduced/increased kidney size [at least one kidney]; 2 = GFR below age range) but did not relate to any other variable. However, the power to identify bivariate correlations concerning SWD levels was just sufficient for large effect sizes (*p* ≥ 0.55, *d* = 1.32). In contrast, analyses regarding ATI and SWE levels were sufficiently powered to identify medium to large effects (*p* ≥ 0.4, *d* = 0.87).

Neither GFR nor pathological kidney function, as defined previously, demonstrated significant associations with biochemical or ultrasound tissue markers of liver structure or pathology. However, a positive correlation was observed between kidney size and increased liver echogenicity (*r*(45) = 0.41, *p* < 0.001) as well as abnormal liver configuration, specifically a rounded/pointed liver margin (*r*(45) = 0.33, *p* = 0.006), suggestive of liver steatosis and fibrosis.

### Multiple regression

Choosing the most appropriate set of covariates by considering the amount of variance explained in ATI, SWE, and SWD measurements against overfitting and the minimum model size, ATI levels in BBS patients were most efficiently determined by liver size (*b* = 0.002, *t*(46) = 4.16, *p* < 0.001) and liver echogenicity (*b* = 0.02, *t*(46) = 5.28, *p* < 0.001, Supplementary Table 1). In contrast, when jointly considering multiple covariates, the only variables to significantly affect SWE and SWD levels were abdominal wall thickness (*b* = 0.01, *t*(47) = 5.50, *p* < 0.001) and sex (*b* = − 1.42, *t*(37) = − 2.17, *p* = 0.04), respectively (Supplementary Table 2 and 3). However, the latter failed to reach significance when considering a correction for multiple comparisons. These analyses were sufficiently powered to detect medium to large effect sizes (*d* = 0.82, assuming up to 11 covariates).

Importantly, independent sample t-tests revealed no significant difference in ATI (*t*(47) = 1.55, *p* = 0.012), SWE (*t*(47) = − 0.68, *p* = 0.25), and SWD (*t*(37) = 0.50, *p* = 0.99) levels between patients fasting less than and more than 2 h and this also applied when considering the covariates identified in the previous step of analysis by ANCOVAs (ATI: (*b* = − 0.145, *t*(44) = − 1.57, *p* = 0.12) | SWE: (*b* = 0.05, *t*(46) = 1.24, *p* = 0.22) | SWD: (*b* = 0.23, *t*(36) = 0.32, *p* = 0.75).

### Group comparisons—BBS subtypes

Comparing the two BBS genotypes with reasonable numbers of affected patients (i.e., type 1 and type 10; N_type1_ = 12, N_type10_ = 16, Table 4) by ANCOVAs, there was no difference regarding ATI (*b* = 0.001, *t*(24) = 0.39, *p* = 0.70), SWE (*b* = − 0.001, *t*(25) = − 0.20, *p* = 0.84), and SWD levels (*b* = − 0.06, *t*(25) = − 0.60, *p* = 0.56) when considering covariates identified by the selection process and its results outlined above. However, ATI levels appeared descriptively higher in the *BBS10* gene group, in particular compared to the *BBS1* hotspot pathogenic variant c.1169T > G p.(Met390Arg) (0.71 vs. 0.56 dB/cm/Mhz). Moreover, further exploratory analyses revealed that *BBS1* patients were taller (height-SDS: *BBS1*: 0.93 (1.22) | *BBS10*: − 0.09 (1.15), p = 0.03) and had higher portal vein flow (*BBS1*: 31.3 (8.63) cm/s | *BBS10*: 25.0 (4.84) cm/s, *p* = 0.047) than *BBS10* patients. In contrast, *BBS10* patients had higher BMI-SDS (*BBS1*: 2.02 (1.40)| *BBS10*: 3.19 (0.97), *p* = 0.02), and there was a statistical trend for increased liver echogenicity (*BBS1*: 2/12 (16.7%) | *BBS10*: 9/16 (56.3%), *p* = 0.05), increased kidney echogenicity (*BBS1*: 3/12 (25%) | *BBS10*: 10/16 (62.5%), *p* = 0.07) and a higher incidence of GFR levels below 90 ml/min (*BBS1*: 1/11 (9.1%) | *BBS10*: 7/15 (46.7%), *p* = 0.08) than *BBS1* patients. These analyses were sufficiently powered to detect findings with effect sizes larger than *d* = 1.28 (assuming up to 3 covariates). The other BBS subgroups comprised between 1 and 4 patients each, rendering the dataset inadequate for robust statistical analysis. Consequently, and instead, descriptive results concerning anthropometric parameters, as well as renal and hepatic findings, in these subgroups are detailed in Table 4.

**Table 4:** Detailed study cohort information according to BBS genes

ID	Age (years)	Sex	Gen	H/C	Pathogenic variation and predicted effect	Height SDS	Weight SDS	BMI SDS	GFR (ml/min/ 1.73m^2^)	Kidney Size (%)	Kidney Structure	Kidney Cyst	persistent fetal lobulation	Liver Size (%)	ATI (dB/cm /Mhz)	SWE (kPa)
		**BBS 1 (n = 12)**														
	**21.2** ± 12.3	**F 50%** **M 50%**	All BBS 1 patients			**0.93** ± 1.17	**1.85** ± 1.58	**2.02** ± 1.34	**8.3%** **reduced**	**102.8** ± 29.9	**66.7%** **abnormal**	**25%** **present**	**66.7%** **present**	**109.1** ± 17.3	**0.62** ± 0.12	**6.3** ± 1.8
	**27.3** ± 11.5	**F 86%** **M 14%**	Only BBS 1 patients with homozygote c.1169 T > G p.(Met390Arg) mutation (n = 7)			**0.55** ± 1.01	**1.07** ± 1.57	**1.50** ± 1.52	**14.3%** **reduced**	**101.1** ** ± 28.5**	**71.4% abnormal**	**28.6%** **present**	**57.1%** **present**	**102.1** ± 12.3	**0.56** ± 0.08	**6.8** ± 2.0
**1**	11.4	M	1	C	c.784_793dup (p.Asn269GlyfsX95) + c.1431_1447del (p.Leu478ArgfsX17)	0.34	2.79	2.88	> 90	79	1	0	1	153	0.91	7
**11**	43	M	1	H	c.1169 T > G (p.Met390Arg)	− 0.40	− 0.24	0.30	> 90	127	1	1	0	86	0.53	6.1
**14**	25	M	1	C	c.1169 T > G (p.Met390Arg) + c.670G− > A (pGlu224− Lys)	2.00	2.44	2.27	> 90	150	0	0	1	114	0.66	5
**18**	11.3	M	1	C	c.1123dupA (p.Ser375Lysfs*40) + c.1169 T > G (p.Met390Arg)	3.04	3.18	2.60	> 90	120	1	1	1	106	0.61	4.8
**20**	13.9	M	1	C	c.1135− 1136delGG (p.Gly379ProfsX35) + c.1169 T > G (p.Arg390Met) *secondary:* *ALMS1 gene c.1267G* > *A (p.VAL423Ile)*	− 0.11	2.07	2.43	> 90	115	0	0	0	120	0.74	5.2
**21**	21	F	1	H	c.1169 T > G (p.Met390Arg)	0.72	1.84	2.62	77	68	0	0	1	99	0.48	5.6
**24**	1.8	M	1	C	c.724− 1G > C + c.1285C > T (p.Arg429*)	2.05	4.17	3.56	> 90	61	1	0	1	101	0.55	5.5
**32**	35	F	1	H	c.1169 T > G (p.Met390Arg) *secondary* *EYS− gene c.4519A* > *G (p.Ile1507Val) (heterozygous; OMIM 602772 Retinitis pigmentosa*)	− 1.44	− 2.09	− 1.22	> 90	91	1	1	0	117	0.53	5.4
**39**	10.8	F	1	H	c.1169 T > G (p.Met390Arg)	1.59	2.3	2.07	> 90	58	1	0	1	88	0.6	7.5
**40**	13	F	1	H	c.1169 T > G (p.Met390Arg)	0.86	2.16	2.04	> 90	101	1	0	0	97	0.64	7.3
**48**	37	F	1	H	c.1169 T > G (p.Met390Arg)	1.19	0.97	0.93	> 90	126	0	0	1	111	0.46	4.7
**49**	31	F	1	H	c.1169 T > G (p.Met390Arg)	1.34	2.57	3.75	> 90	137	1	0	1	118	0.68	11.2
		**BBS 2 (n = 2)**														
	**19.9** ± 1.9	**F 50%** **M 50%**				**− 0.89** ± 0.08	**1.11** ± 0.09	**1.95** ± 0.05	**50%** **reduced**	**141**	**100%** **abnormal**	**0%** **present**	**0%** **present**	**95.3** ± 3.7	**0.60** ± 0.01	**6.3** ± 1.40
**34**	18	F	2	C	c.823C > T (p.Arg275*) + c.1986dupT (p.Asn663*)	− 0.96	1.02	1.99	< 10 (ESKD at 6 years)	Renal transplantation + graft failure and following dialysis				99	0.6	4.9
**38**	22	M	2	C	c.823C > T (p.Arg275) + c.1986dupT (p.Asn663)	− 0.81	1.20	1.90	> 90	141	1	0	0	92	0.59	7.7
		**BBS 4 (n = 3)**														
	**13.6** ± 5.3	**F 33.3%** **M 66.7%**				**− 1.49** ± 0.66	**1.67** ± 0.71	**2.59** ± 0.51	**33.3%** **reduced**	**105.5** ± 47.0	**66.7%** **abnormal**	**33.3%** **present**	**66.7%** **present**	**118.6** ± 8.2	**0.77** ± 0.04	**5.9** ± 0.7
**3**	6.2	M	4	H	c.(76 + 1_77− 1)_(220 + 1_221− 1)del (p.Pro27_Ala74del)	− 1.75	0.73	2.08	73	49	1	1	0	129	0.71	4.9
**28**	16.8	F	4	H	c.210_213delCTA (p.Ile70Metfs*5)	− 2.14	2.44	3.29	> 90	103	1	0	1	109	0.8	6.2
**29**	18	M	4	H	deletion exon 7 and 8	− 0.58	1.83	2.40	> 90	164	0	0	1	118	0.8	6.5
		**BBS 5 (n = 3)**														
	**6.6** ± 2.4	**F 66.7%** **M 33.3%**				**− 0.63** ± 0.38	**1.09** ± 0.48	**1.83** ± 0.49	**0%** **reduced**	**107.9** ± 28.7	**100%** **abnormal**	**0%** **present**	**33.3%** **present**	**103.7** ± 7.3	**0.63** ± 0.04	**4.7** ± 0.7
**25**	6.4	F	5	C	c.54dupC p. (ala19Argfs*14) + deletion Exon 10–12	− 0.34	1.77	2.38	> 90	148	1	0	0	113	0.67	5.2
**26**	9.6	F	5	C	c.54dupC p. (ala19Argfs*14) + deletion Exon 10–12	− 0.37	0.77	1.19	> 90	85	1	0	0	95	0.57	5.2
**27**	3.7	M	5	C	c.54dupC p. (ala19Argfs*14) + deletion Exon 10–12	− 1.17	0.74	1.92	> 90	91	1	0	1	103	0.65	3.8
		**BBS 7 (n = 2)**														
	**19.0** ± 3.0	**F 100%** **M 0%**				**0.05** ± 0.10	**2.97** ± 0.63	**3.63** ± 0.16	**0%** **reduced**	**141.7** ± 33.3	**50%** **abnormal**	**0%** **present**	**50%** **present**	**118.6** ± 9.1	**0.68** ± 0.04	**8.3** ± 0.8
**9**	16	F	7	H	c.968A > G (p.His323Arg)	0.14	3.59	3.47	> 90	108	0	0	0	110	0.72	9.1
**10**	22	F	7	C	c.1886 T > A (p.Leu629*) + c.712_715del p.(Arg238Glufs*59)	− 0.05	2.34	3.78	> 90	175	1	0	1	128	0.64	7.5
		**BBS 8 (n = 4)**														
	**7.5** ± 4.5	**F 50%** **M 50%**				**0.94** ± 0.59	**2.91** ± 0.66	**2.93** ± 0.37	**25%** **reduced**	**112.7** ± 26.6	**75%** **abnormal**	**0%** **present**	**50%** **present**	**128.1** ± 24.7	**0.73** ± 0.11	**6.4** ± 1.9
**5**	10.8	F	8	H	deletion Exon 9	1.80	3.63	3.28	> 90	154	1	0	1	158	0.8	5
**22**	3.7	M	8	H	c.776A > G (p.Asp259Gly)	0.99	3.37	3.28	75	88	1	0	0	109	0.64	5.5
**23**	2.5	F	8	H	c.776A > G (p.Asp259Gly)	0.16	1.91	2.42	> 90	90	0	0	1	99	0.61	5.3
**47**	12.8	M	8	H	c.915delG (p.Met305fs)	0.79	2.73	2.72	> 90	119	1	0	0	147	0.86	9.7
		**BBS 9 (n = 1)**														
**31**	22	F	9	H	c.263 + 1G > P (IVS3 + 1G > P)	**− 2.05**	**0.69**	**2.27**	** > 90**	**76**	**1**	**0**	**0**	**92**	**0.55**	**4.9**
		**BBS 10 (n = 16)**														
	**20.0** ± 12.6	**F 31%** **M 69%**				**− 0.09** ± 1.11	**2.65** ± 1.19	**3.19** ± 0.94	**43.8%** **reduced**	**126.5** ± 46.4	**75%** **abnormal**	**25%** **present**	**31.3%** **present**	**119.8** ± 18.6	**0.71** ± 0.09	**6.6** ± 1.4
**2**	13.8	F	10	C	c.271dupT (p.Cys91LeufsX5) + c.273C < G (p.Cys91Trp)	0.07	2.80	2.85	> 90	87	1	0	0	111	0.58	5.2
**6**	14.3	M	10	H	c.1269_1273del (p.Gln423fsX)	− 1.08	3.92	3.70	> 90	177	1	0	0	152	0.79	6.6
**7**	51	F	10	C	c.1448_1452del (p.Thr483Asnfs*8) + c.271dup (p.Cys91Leufs*5)	0.26	1.95	2.99	16 (dialysis; ESKD at 46 years)	89	1	0	0	152	0.7	8.1
**8**	29	F	10	C	c.1330dup (p.Ser444Lysfs*6) + c.1555_1564del (p.Thr519Argfs*2)	1.03	2.90	4.53	> 90	184	1	0	1	124	0.61	8.9
**12**	12.7	M	10	H	c.271dupT (p.Cys91Leufs*5)	0.52	3.28	3.16	31	123	1	1	0	141	0.75	6.2
**13**	18	M	10	C	c.145CA > T (p.Arg49Trp) + c.271dupT (p.Cys91LeufsX5)	− 0.49	1.77	2.35	46	57	1	0	1	95	0.58	7
**15**	24	M	10	C	c.271dupT (p.Cys91Leufs*5) + c.235dupA (p.Thr79Asnfs*17)	− 1.10	1.06	2.00	> 90	87	0	0	0	125	0.83	7
**17**	22	M	10	C	c.145C > T (p.Arg49Trp) + c.680_681delinsTT (p.Gly227Val)	0.86	2.10	2.29	> 90	105	0	1	1	99	0.75	5
**19**	41	M	10	H	c.271dupT (p.Cys91Leufs*5)	− 0.26	2.90	3.38	72	200	1	1	0	128	0.73	7.1
**33**	17.6	M	10	H	c.271dupT (p.Cys91Leufs*5)	− 0.20	2.84	2.80	88	129	1	1	0	99	0.76	4.9
**36**	2.3	M	10	H	c. 154del (p.Thr516Asnfs*8)	1.85	4.89	4.07	> 90	70	1	0	0	127	0.64	5.4
**41**	27	F	10	C	c.271dupT (p.Cys91Leufs*5) + c.273C > G (p.Cys91Trp)	− 0.98	2.7	4.69	> 90	143	0	0	0	109	0.76	9.8
**42**	19	M	10	H	c.1269_1273del (p.Gln423fsX)	− 1.78	2.89	3.71	72	174	1	0	0	139	0.9	7.5
**43**	21	M	10	C	c.164 T > C (p.Leu55Pro) + c.986C > T (p.Ser329 Leu)	− 2.20	− 0.33	1.23	> 90	152	0	0	1	97	0.6	5.7
**44**	6.9	M	10	C	c.271dupT (p.Cys91Leufs*5) + c.1599_1602delAACT (p.Thr534Ilefs*21)	0.62	2.57	2.85	> 90	182	1	0	1	102	0.62	5.6
**46**	1.1	F	10	H	c.271dupT (pCys91Leufs*5)	1.52	4.14	4.43	63	64	1	0	0	117	0.68	5.0
		**BBS 12 (n = 4)**														
	**19.4** ± 12.3	**F 75%** **M 25%**				**0.42** ± 0.91	**2.03** ± 1.31	**2.69** ± 1.53	**0%** **reduced**	**123.1** ± 58.5	**75%** **abnormal**	**50%** **present**	**50%** **present**	**104.8** ± 12.1	**0.69** ± 0.11	**5.8** ± 0.7
**4**	4.9	F	12	C	c.1483_1484delGA (p.Glu495Argfs*3) + c.1573_1574insT (p.Arg525Leufs*19)	1.93	3.70	3.61	> 90	87	1	1	0	86	0.6	5.3
**16**	36	M	12	C	c.356G > A (p.Gly119Asp) + c.1115_1116delTT (p.Phe372X)	− 0.12	1.00	1.51	> 90	86	1	0	0	103	0.58	6.8
**35**	26	F	12	H	c.1375C > T (p.Gln459*)	0.26	2.89	4.71	> 90	224	0	1	1	118	0.84	6.1
**45**	10.7	F	12	C	c.1115_1116del (p.F372fs) + c.476C > T (p.P159L)	− 0.41	0.53	0.93	> 90	96	1	0	1	113	0.74	4.9
		**BBS 16 (n = 1)**														
**30**	17.3	F	16	H	c.985dup (p.Thr329Asnfs*10) *secondary* *PKD2 gene; heterogenous* *c.868G* > *A (p.Gly290Arg)*	**− 3.01**	**3.1**	**4.06**	**63** **(ESKD at 10 years)**	**Renal transpl**	**1**	**0**	**0**	**135**	**0.87**	**6.1**
		**BBS 17 (n = 1)**														
**37**	11	F	17	H	c.778–3 C > T *further information:* *Localization at the transition from intron 7 to exon 8 in the LZTFL1 gene (BBS 17; OMIM #615,994); impairment/loss of the corresponding splice donor site, variant of unclear significance*	**− 1.06**	**0.05**	**0.75**	**84**	**76**	**1**	**0**	**0**	**110**	**0.70**	**5.2**

Averaged results of all patients with pathogenic variants in the same BBS gene are indicated in bold

Further information: Siblings ID22 + 23; ID 25 + 26 + 27; ID39 + 40; ID 6 + 42; ID 48 + 49

F—female, M—male, C—compound heterozygous, H—homozygous

### Group comparisons—BBS vs. norming sample

In contrast, an ANCOVA, considering liver size, liver echogenicity, age, and BMI-SDS, revealed higher ATI levels in the BBS than the norming sample (*b* = 0.07, *t*(151) = 4.60, *p* < 0.001) beyond the age of 2 years as investigated by a Neyman-Johnson analysis for reasons of heterogeneity of regression slopes (interaction group x age: *b* = 0.004, *t*(151) = 2.84, *p* = 0.005; for details, please see the Supplementary Material and Supplementary Fig. 1). In addition, SWE levels were found to be higher in the BBS than the norming sample (*b* = 0.07, *t*(155) = − 2.85, *p* = 0.005) when considering an analysis-specific subset of covariates (abdominal wall thickness, age, and BMI-SDS) and a heteroscedasticity consistent standard-error estimator (HC3). These results did not change when excluding a single outlier concerning the analyses of ATI (*b* = 0.07, *t*(150) = 4.54, *p* < 0.001) and SWE levels ((*b* = 0.07, *t*(154) = − 2.61, *p* = 0.01)), including sex, age, and BMI-SDS as covariates. No difference was observed in SWD measurements (b = − 0.85, *t*(136) = − 1.64, *p* = 0.10). These findings have to be interpreted against sufficient power to identify medium-sized effects (*d* = 0.64) (Table 5).

**Table 5:** Bivariate correlations of the new ultrasound techniques ATI, SWE, and SWD and potential influencing variables in BBS

	ATI (dB/cm/MHz)	SWE (ms)	SWD (m/s/kHz)	age (years)	sex	BMI-SDS	height-SDS	abdominal wall (mm)	Coop eration	fasting duration	live size (%)	liver echo genicity	liver margin	Gall blader state	A. hepatica (cm/s)	V. hepatica (cm/s)	V. portae (cm/s)	GOT	GPT	GGT	AP	spleen size (%)	kidney function	kidney size (%)	kidney pathology	GFR
ATI (dB/cm/MHz)	1,00	0,15	0,15	− 0,02	− 0,14	0.31^**^	− 0,13	0.35^**^	0,02	− 0,21	0.48^**^	0.60^**^	0,17	− 0,05	0,07	− 0,09	0,03	0,22	0.33^**^	0.29^**^	0,07	0.27^*^	0,14	0.25^*^	0,04	0,11
SWE (ms)	0,15	1,00	0,14	0.27^**^	0,02	0.25^*^	− 0,04	0.35^**^	0,23	0,11	0,14	0,20	0,05	0,04	0,12	− 0,06	− 0,03	− 0,08	0,11	0.22^*^	− 0,13	0,11	− 0,09	0,15	− 0,12	− 0,08
SWD (m/s/kHz)	0,15	0,14	1,00	0,07	− 0,23	0,01	− 0,07	0,06	0,20	− 0,01	0,11	0,11	0,10	0,06	− 0,08	0,16	− 0,01	0,06	− 0,03	− 0,08	− 0,06	0,14	− 0,25	0,05	− 0.37^**^	− 0,27
age (years)	− 0,02	0.27^**^	0,07	1,00	0,03	− 0,08	− 0,16	0.20^*^	0.41^**^	0,06	0,03	0,14	0,09	− 0,06	0,00	− 0,07	− 0.22^*^	− 0.30^**^	− 0,12	0,18	− 0.51^**^	0,13	0,01	0.28^**^	− 0,02	0,00
sex	− 0,14	0,02	− 0,23	0,03	1,00	0,09	0,03	0,06	0,18	0,10	− 0,07	− 0.32^*^	− 0,07	0,06	0,17	− 0,06	0.33^*^	− 0.28^*^	− 0,25	0,10	− 0,19	− 0.32^**^	− 0,12	− 0,08	− 0,08	− 0,10
BMI-SDS	0.31^**^	0.25^*^	0,01	− 0,08	0,09	1,00	0.21^*^	0.41^**^	− 0,04	− 0,10	0.28^**^	0.29^*^	0,21	0,04	0,18	− 0,13	0,05	0,09	0,17	0.27^*^	− 0,06	0,03	0,08	0,20	0,10	0,08
height-SDS	− 0,13	− 0,04	− 0,07	− 0,16	0,03	0.21^*^	1,00	0,12	− 0,03	− 0.24^*^	0,00	− 0,04	0,05	− 0,08	0,01	0,16	0,08	0.24^*^	0,11	0,10	0,17	− 0.25^*^	− 0,13	− 0,05	− 0,06	− 0,14
abdominal wall (mm)	0.35^**^	0.35^**^	0,06	0.20^*^	0,06	0.41^**^	0,12	1,00	0,21	− 0,07	0.25^*^	0.42^**^	0.28^*^	0,07	0.27^*^	− 0.26^*^	− 0,02	0,05	0.27^*^	0.36^**^	− 0,03	0,11	0,02	0.35^**^	− 0,03	0,01
ccoperation	0,02	0,23	0,20	0.41^**^	0,18	− 0,04	− 0,03	0,21	1,00	− 0,13	0,01	0,18	0,14	− 0,19	0,19	− 0,01	0,03	− 0.30^*^	− 0,14	0,14	− 0,15	0,03	− 0,13	0,23	− 0,10	− 0,14
fasting state	− 0,21	0,11	− 0,01	0,06	0,10	− 0,10	− 0.24^*^	− 0,07	− 0,13	1,00	− 0,19	− 0,20	− 0,23	0.51^**^	0,19	0,07	0,07	− 0,05	− 0,17	− 0,21	0,09	− 0,05	0,02	− 0,12	0,04	0,04
live size (%)	0.48^**^	0,14	0,11	0,03	− 0,07	0.28^**^	0,00	0.25^*^	0,01	− 0,19	1,00	0.37^**^	0.32^**^	− 0,08	− 0,10	− 0,11	− 0,11	0,21	0.24^*^	0.23^*^	0,01	0,13	0,13	0.20^*^	0,12	0,11
liver echogenicity	0.60^**^	0,20	0,11	0,14	− 0.32^*^	0.29^*^	− 0,04	0.42^**^	0,18	− 0,20	0.37^**^	1,00	0,18	− 0,08	0,14	− 0,10	− 0,12	0,23	0.47^**^	0.41^**^	0,01	0.27^*^	0,10	0.41^**^	0,06	0,05
liver margin	0,17	0,05	0,10	0,09	− 0,07	0,21	0,05	0.28^*^	0,14	− 0,23	0.32^**^	0,18	1,00	− 0,15	− 0,06	− 0,21	− 0,09	0,02	0,18	0,17	0,04	0,09	− 0,07	0.33^**^	− 0,07	− 0,06
gallblader state	− 0,05	0,04	0,06	− 0,06	0,06	0,04	− 0,08	0,07	− 0,19	0.51^**^	− 0,08	− 0,08	− 0,15	1,00	0,16	0,08	0,04	0,20	0,02	− 0,10	0,07	− 0,04	− 0,08	0,06	0,08	− 0,07
A. hepatica (cm/s)	0,07	0,12	− 0,08	0,00	0,17	0,18	0,01	0.27^*^	0,19	0,19	− 0,10	0,14	− 0,06	0,16	1,00	− 0,13	0,09	0,06	0,12	0,25	− 0,10	0,00	− 0,27	0,08	− 0,17	− 0,22
V. hepatica (cm/s)	− 0,09	− 0,06	0,16	− 0,07	− 0,06	− 0,13	0,16	− 0.26^*^	− 0,01	0,07	− 0,11	− 0,10	− 0,21	0,08	− 0,13	1,00	0,06	0,23	− 0,03	− 0,10	0,04	− 0,08	− 0,04	− 0,08	− 0,03	− 0,04
V. portae (cm/s)	0,03	− 0,03	− 0,01	− 0.22^*^	0.33^*^	0,05	0,08	− 0,02	0,03	0,07	− 0,11	− 0,12	− 0,09	0,04	0,09	0,06	1,00	− 0,01	− 0,20	0,01	0,10	− 0,05	− 0,01	− 0,03	0,00	0,02
GOT (U/l)	0,22	− 0,08	0,06	− 0.30^**^	− 0.28^*^	0,09	0.24^*^	0,05	− 0.30^*^	− 0,05	0,21	0,23	0,02	0,20	0,06	0,23	− 0,01	1,00	0.39^**^	0.23^*^	0.26^*^	− 0,10	− 0,03	0,06	0,08	− 0,03
GPT (U/l)	0.33^**^	0,11	− 0,03	− 0,12	− 0,25	0,17	0,11	0.27^*^	− 0,14	− 0,17	0.24^*^	0.47^**^	0,18	0,02	0,12	− 0,03	− 0,20	0.39^**^	1,00	0.39^**^	0,16	0,07	− 0,03	0.28^*^	0,06	− 0,03
GGT (U/l)	0.29^**^	0.22^*^	− 0,08	0,18	0,10	0.27^*^	0,10	0.36^**^	0,14	− 0,21	0.23^*^	0.41^**^	0,17	− 0,10	0,25	− 0,10	0,01	0.23^*^	0.39^**^	1,00	− 0,13	0,01	0,04	0.37^**^	0,11	0,04
AP (U/l)	0,07	− 0,13	− 0,06	− 0.51^**^	− 0,19	− 0,06	0,17	− 0,03	− 0,15	0,09	0,01	0,01	0,04	0,07	− 0,10	0,04	0,10	0.26^*^	0,16	− 0,13	1,00	− 0,04	0,01	− 0,15	0,02	0,01
spleen size (%)	0.27^*^	0,11	0,14	0,13	− 0.32^**^	0,03	− 0.25^*^	0,11	0,03	− 0,05	0,13	0.27^*^	0,09	− 0,04	0,00	− 0,08	− 0,05	− 0,10	0,07	0,01	− 0,04	1,00	0,15	0,07	0,07	0,14
kidney function	0,14	− 0,09	− 0,25	0,01	− 0,12	0,08	− 0,13	0,02	− 0,13	0,02	0,13	0,10	− 0,07	− 0,08	− 0,27	− 0,04	− 0,01	− 0,03	− 0,03	0,04	0,01	0,15	1,00	− 0,20	0.85^**^	1.00^**^
kidney size (%)	0.25^*^	0,15	0,05	0.28^**^	− 0,08	0,20	− 0,05	0.35^**^	0,23	− 0,12	0.20^*^	0.41^**^	0.33^**^	0,06	0,08	− 0,08	− 0,03	0,06	0.28^*^	0.37^**^	− 0,15	0,07	− 0,20	1,00	− 0,08	− 0,19
kidney pathology	0,04	− 0,12	− 0.37^**^	− 0,02	− 0,08	0,10	− 0,06	− 0,03	− 0,10	0,04	0,12	0,06	− 0,07	0,08	− 0,17	− 0,03	0,00	0,08	0,06	0,11	0,02	0,07	0.85^**^	− 0,08	1,00	0.84^**^
GFR	0,11	− 0,08	− 0,27	0,00	− 0,10	0,08	− 0,14	0,01	− 0,14	0,04	0,11	0,05	− 0,06	− 0,07	− 0,22	− 0,04	0,02	− 0,03	− 0,03	0,04	0,01	0,14	1.00^**^	− 0,19	0.84^**^	1,00

The table displays Kendall’s τ for reasons outlined in the Methods section of the manuscript. Fasting state (0 < 2 h, 1 >  = 2 h) and kidney function (0 >  = 90 ml/min, 1 < 90 ml/min) were dichotomized for correlation analysis. Liver echogenicity: 0 = normal, 1 = increased; liver margin: 0 = concave; 1 = pointed, 2 = rounded. Liver and spleen size is given in % of normal values adjusted for body height and age. AP, alkaline phosphatase; ATI, attenuation index; BMI, body mass index; GGT, gamma-glutamyltransferase; GOT, glutamic-oxaloacetic transaminase; GPT, glutamic-pyruvic transaminase; SDS, standard deviation score; SWD, shear wave dispersion; SWE, shear wave elastography. *p < .05 (bold type); **p < .01 (bold type and italic)

## Discussion

We detected anomalies of the parenchyma of the kidney and liver in a substantial proportion of our BBS cohort by using high-resolution US together with emerging technical devices (ATI, SWE, and SWD), enabling the quantitative and qualitative assessment of liver tissue in particular. The percentage of patients with parenchymal alterations was significantly higher than reported from other studies, and those were detected at an early stage—in part even before the elevation of liver enzymes—indicating a high sensitivity of the applied methods.

### Study cohort

The distribution of pathogenic variants in our BBS cohort was similar to other recent studies from Europe and the USA with a predominance of pathogenic variations in *BBS1* and *BBS10* [9–11], but differed from a study in China, where the majority of patients were affected by mutations in *BBS2* and *BBS7* [42]. Moreover, we noted an accumulation of the hot spot mutations *c.271dupT* (p.Cys91Leufs*5) in *BBS10* and *c.1169 T* > *G* (p.Met390Arg) in *BBS1.* The proportion of two truncating mutations was higher than the subgroup of two missense mutations and thus different from other recent studies [9, 10]. Overall, BMI SDS was increased in our patients (with a wide range) and significantly higher in patients with mutations in *BBS10* compared to those with *BBS1* mutations, consistent with published data [13]. Five patients (10%) presented with a BMI SDS <  + 1SD, three of these had pathogenic variants in the *BBS1* gene, concomitant with an overall less severe phenotype. The other two patients were 11-year-old children with pathogenic variants in *BBS12* and *BBS17*, respectively. Given the rarity of clinical cases involving *BBS17*, it remains unclear whether this subtype in general also exhibits a tendency towards a milder obesity. Body length was within the normal range without any differences regarding sex and age. However, the subgroup of *BBS1* patients was significantly taller than patients affected by a pathogenic variation in *BBS10.* This height difference may be determined by several factors. Notably, *BBS1* patients often exhibit milder renal involvement, which typically correlates with improved growth potential [11, 13]. Additionally, *BBS1* patients tend to display an overall less severe phenotype compared to individuals with other BBS types [7, 11, 13]. This suggests a less profound impairment of metabolic pathways and hormonal signaling cascades in *BBS1* patients, which could contribute to the observed difference in body length.

### Renal involvement

In children, the average total kidney volume fell within the normal range, albeit with considerable variation. The higher volumes observed in adults could be attributed to the correlation between renal size and BMI [43, 44] and the fact that normal values were adjusted for weight up to a limit of 60 kg, a threshold significantly exceeded by the adult patients. Kidney abnormalities were detected in 75% of patients, which is a substantially higher proportion than in other studies [1, 2, 9, 11]. Persistent fetal lobulation was present in 44% and therefore more than 10 times as frequent compared to the estimated prevalence (4%) in the normal population. This may indicate impaired embryonic renal development as a result of the underlying ciliopathy in BBS [45]. In the literature, patients with variants in the BBS genes 2, 5, 8, 9, 10, 12, 16, and 17 were demonstrated to develop a more severe kidney phenotype than patients with mutations in *BBS1*, *BBS4*, or *BBS7*. This was comparable to our study. However, in our study, the latter subgroups also showed a multitude of structural anomalies with a higher frequency than in other studies [42], indicating the high sensitivity of the applied US methods [46]. Consistent with recent studies [9–11], our results support the milder renal involvement in patients with *BBS1* mutations.

The RI was elevated in 55% of cases, indicating pathology of the renal parenchyma. Microperfusion imaging may allow a more precise evaluation of parenchymal changes in the kidney prospectively. However, to date, standardized quantification methods are lacking, especially in the case of obesity [47].

Urinary tract disorders were rarely detected in our cohort. However, a high bladder volume, measured incidentally, was seen in 20% of cases. This finding should be considered during clinical evaluations and when assessing possible post-renal complications [12, 40].

Twenty-seven percent of patients presented with an impaired kidney function (CKD 2–5), 23% of these (6% of the total study population) progressed to ESKD. These numbers are comparable to other studies or even lower, especially considering the substantial proportion of adult patients in our cohort given. Impairment of renal function was a particular finding in patients with a pathogenic variant in *BBS10* (and, to a lesser extent, in those with variants in BBS genes *2, 4, 16,* and *17*). Conversely, patients with variants in *BBS1, 5, 7, 9,* and *12* presented with stable kidney function. The three patients who developed ESKD were affected by mutations in *BBS2*, *10,* and *16* and started renal replacement therapy at ages 6, 10, and 46 years. Recent data indicate that the risk for the development of ESKD is not only increased in the first year of life and at preschool age [9], but also later in adulthood, with a higher prevalence in females than in males [10]. The inherited structural abnormalities of the kidneys in BBS, when combined with the metabolic risks associated with obesity and secondary conditions such as type 2 diabetes or MASLD, may serve as an additional factor exacerbating the decline in kidney function later in life [48].

### Hepatic involvement

Over half of the cohort demonstrated ATI and SWE levels above the 97th percentile, indicative of hepatic parenchymal changes such as steatosis and fibrosis. This exceeds even the high figures reported in the literature, which indicates a prevalence of steatosis in 27% of adult BBS patients [1, 2, 19]. Accordingly, ATI and/or SWE were altered in a substantial proportion of our patients, although standard US examination (size, echogenicity, and liver margin) as well as serum liver enzymes were within the normal range, indicating a high sensitivity of the applied US techniques and their ability to reveal even subtle parenchymal lesions [23]. ATI showed a weak association with the size of the liver, spleen, and kidney, as well as liver echogenicity and enzymes, all of which are indicators of hepatic steatosis. ATI and SWE were associated with BMI SDS and abdominal wall thickness, obesity-related parameters, and well-known drivers of hepatic steatosis and fibrosis. Again, patients with a *BBS10* mutation were more severely affected (and showed higher ATI levels) than those with a *BBS1* mutation.

SWD was significantly increased (above the 97th percentile) in 23% of our cohort, displaying a higher occurrence among males*.* Elevated SWD levels indicate inflammation and increased liver viscosity [23]. The correlation with the male sex might point to a potential higher inflammation risk within this subgroup. However, the lower incidence of pathological kidney findings indicates that liver viscosity could also be influenced by other variables, underscoring the need for further research [49]. The hepatic phenotype in BBS widely varies from MASLD, including steatosis and Metabolic Dysfunction-associated Steatohepatitis (MASH), to liver cirrhosis and an increased risk of hepatocellular carcinoma [50–52].

Despite these important insights into the ultrasound phenotype of BBS patients, the cross-sectional design of our study does not allow to draw directional conclusions regarding the (relative) contribution of obesity and ciliopathy in BBS to the pathological ATI, SWE, and SWD findings in these patients. However, our findings suggest that severe early-onset obesity is a potential driver of liver involvement in BBS. The contribution of ciliary dysfunction [53] and of a specific hepatic BBSome [54] in this context remains to be determined by future studies, for example drug intervention studies, by decrease of the obesity-related effect. The Melanocortin 4 Receptor (MC4R) agonist (setmelanotide) has already been approved by the European Medicines Agency for the treatment of children aged six and above [55]. MC4R–deficient animals have been established as successful models of MASLD [56–58], and the hepatic expression of the MC4 receptor has been demonstrated [59, 60]. Thus, high-definition US combined with an interventional study could not only help disentangle the contribution of obesity and ciliopathy in BBS but also play an important role in assessing the benefit of new therapeutic options by monitoring therapeutic success.

### Limitations

In a small number of patients, complete diagnostic data could not be obtained due to technical availability (SWD) or technical challenges associated with obesity (liver perfusion). As BBS is a rare disease, the number of participants was limited. However, the present cohort included a larger number of patients compared to previous clinical studies. In addition, the large number of different BBS genes and pathogenic variants within these genes complicates analyses. Unfortunately, histopathological data from liver biopsy were available for only one patient. Therefore, the results from the US examinations, especially concerning the new, high-end technologies, had to be interpreted by comparison with published data from healthy children and adults as well as patients with other underlying diseases [29, 32].

## Conclusion

We evaluated the involvement of the liver and kidney in children and adults with genetically confirmed BBS and the diagnostic potential of high-end ultrasound technologies, including the emerging techniques ATI, SWE, and SWD. Although in part discrete, parenchymal alterations in the liver and kidney were observed in a majority of BBS patients and in a substantially higher proportion than previously described.

Future research involving advanced ultrasound technologies is needed to confirm our findings, elucidating the influence of obesity and ciliopathy on liver disease in BBS, and to quantify the potential impact of weight loss interventions, including GLP-1 analogs and MC4-receptor agonists, on liver involvement and mitigating the risk of end-stage kidney disease (ESKD) in BBS patients.

## Supplementary Information

Additional file 1.

Additional file 2.

Additional file 3.

## Data Availability

The data are available upon reasonable request. All data relevant to the study are included in the article or uploaded as supplementary information. Additional clinical and genetic data are available on reasonable request from the NEOCYST clinical database (www.neocyst.de) and from the corresponding authors.
