# Supplementary material for: Ultrasound evaluation of kidney and liver involvement in Bardet–Biedl syndrome
Source: Orphanet J Rare Dis. 2024 Nov 12;19:425. doi: 10.1186/s13023-024-03400-w (PMC11556208; doi:10.1186/s13023-024-03400-w)
Supplement: Supplementary file 1 — Additional file 1. [file 13023_2024_3400_MOESM1_ESM.docx]

Supplementary Material

Ultrasound evaluation of kidney and liver involvement in Bardet-Biedl syndrome

Metin Cetiner, Ilja Finkelberg, Felix Schiepek, Raphael Hirtz, Anja K. Büscher

**Outline**

Supplementary Methods - Testing of statistical assumptions

Supplementary Results - Table 1 | Covariate Selection Process by FWDselect – ATI

Supplementary Results - Table 2 | Covariate Selection Process by FWDselect – SWD

Supplementary Results - Table 3 | Covariate Selection Process by FWDselect – SWE

Supplementary Results - Figure 1 | Heterogeneity of Regression Slopes

**Supplementary Methods - Testing of statistical assumptions**

Descriptive Statistics

Normality of the data was assessed by the Kolmogorov-Smirnov test. Equality of variances was tested by Levene's test. When this assumption was not met, a Welch test was performed. In case of non-normally distributed data or the presence of outliers, a median test was performed.

Multiple Regression

Normality of the residuals of the multiple regression analyses regarding the outcome measures ATI, SWE, and SWD levels were assessed by the Kolmogorov-Smirnov test. Outlier detection relied on standardized residuals exceeding ­± 3 SD. Multicollinearity was defined by variance inflation factors exceeding 10 and homoscedasticity was evaluated by the Breusch-Pagan test. Autocorrelations of residuals were excluded by the Durbin-Watson test. Linearity was checked by visual inspection of (partial) bivariate scatter plots between the DV of interest and all IVs as well as all pairs of IVs.

ANCOVA

The normality of studentized residuals and the identification of outliers were evaluated using the methods detailed previously in the context of multiple regression analysis. Equality of variances was tested by Levene's test (based on the median). In the presence of heteroscedasticity, a robust standard error estimator for regression coefficients according to Davidson-McKinnan (HC3) was applied. Homogeneity of regression was investigated by separate ANCOVAs testing for the significance of the interaction between the independent (grouping) variable and the considered covariates (1). In the presence of heterogeneity of regression slopes, a Neyman-Johnson analysis was performed. This statistical technique identifies ranges of values for a moderator variable (e.g., age) where the effect of a predictor variable (e.g., group) on an outcome variable (e.g., ATI) is significant. In contexts of heterogeneity of regression slopes, it specifies the range within the moderator variable for which the effect is statistically significant, thus delineating clear boundaries for the influence of the predictor (26).

Supplementary Table 1 - Covariate Selection Process by FWDselect – ATI

|  | **covariate** | | | | | | | | | | |  |  |
| --- | --- | --- | --- | --- | --- | --- | --- | --- | --- | --- | --- | --- | --- |
| **q** | **age** | **sex** | **height-SDS** | **BMI-SDS** | **weight-SDS** | **abdominal wall** | **liver size (%)** | **liver echogenicity** | **liver margin** | **fasting duration** | **cooperation** | **AIC** | **p-value** |
| 1 |  |  |  |  |  |  |  | x |  |  |  | -108.62 | .02 |
| 2 |  |  |  |  |  |  | x | x |  |  |  | -122.29 | .16 |
| 3 |  |  | x |  |  |  | x | x |  |  |  | -126.03 |  |
| 4 | x |  | x |  |  |  | x | x |  |  |  | -131.99 |  |
| 5 | x |  | x |  |  | x | x | x |  |  |  | -134.63 |  |
| 6 | x |  | x |  |  | x | x | x |  | x |  | -135.00 |  |
| 7 | x |  | x |  |  | x | x | x | x | x |  | -136.35 |  |
| 8 | x |  | x | x |  | x | x | x | x | x |  | -135.04 |  |
| 9 | x | x | x | x |  | x | x | x | x | x |  | -133.25 |  |
| 10 | x | x | x | x | x | x | x | x | x | x |  | -131.37 |  |
| ATI FWDselect results. q = size of the considered covariate subset; x = covariate selected by the FWDselect algorithm at size q; AIC = Akaike information criterion; p-value = p-value regarding the hypothesis H1(q): the model fit improves including additional covariates. | | | | | | | | | | | | | |

Supplementary Table 2 - Covariate Selection Process by FWDselect – SWE

|  | **covariate** | | | | | | | | | | |  |  |
| --- | --- | --- | --- | --- | --- | --- | --- | --- | --- | --- | --- | --- | --- |
| **q** | **age** | **sex** | **height-SDS** | **BMI-SDS** | **weight-SDS** | **abdominal wall** | **liver size (%)** | **liver echogenicity** | **liver margin** | **fasting duration** | **cooperation** | **AIC** | **p-value** |
| 1 |  |  |  |  |  | x |  |  |  |  |  | -62.61 | .52 |
| 2 | x |  |  |  |  | x |  |  |  |  |  | -63.71 |  |
| 3 | x |  |  | x |  | x |  |  | x |  |  | -63.79 |  |
| 4 | x |  |  | x |  | x |  |  | x |  |  | -63.16 |  |
| 5 | x |  |  | x |  | x |  |  | x |  | x | -62.03 |  |
| 6 | x |  |  | x |  | x | x |  | x |  | x | -60.60 |  |
| 7 | x |  |  | x |  | x | x | x | x |  | x | -59.32 |  |
| 8 | x |  |  | x | x | x | x | x | x |  | x | -57.77 |  |
| 9 | x | x |  | x | x | x | x | x | x |  | x | -56.20 |  |
| 10 | x | x |  | x | x | x | x | x | x | x | x | -54.38 |  |
| SWE (ms) FWDselect results. q = size of the considered covariate subset; x = covariate selected by the FWDselect algorithm at size q; AIC = Akaike information criterion; p-value = p-value regarding the hypothesis H1(q): the model fit improves including additional covariates. | | | | | | | | | | | | | |

Supplementary Table 3 - Covariate Selection Process by FWDselect – SWD

|  | **covariate** | | | | | | | | | | |  |  |
| --- | --- | --- | --- | --- | --- | --- | --- | --- | --- | --- | --- | --- | --- |
| **q** | **age** | **sex** | **height-SDS** | **BMI-SDS** | **weight-SDS** | **abdominal wall** | **liver size (%)** | **liver echogenicity** | **liver margin** | **fasting duration** | **cooperation** | **AIC** | **p-value** |
| 1 |  | x |  |  |  |  |  |  |  |  |  | 170.14 | .91 |
| 2 |  | x |  |  |  |  |  |  |  |  | x | 170.00 |  |
| 3 |  | x |  |  | x |  |  |  |  |  | x | 170.77 |  |
| 4 |  | x |  | x | x |  |  |  |  |  | x | 170.31 |  |
| 5 | x | x |  | x | x |  |  |  |  |  | x | 171.08 |  |
| 6 | x | x |  | x | x |  | x |  |  |  | x | 171.55 |  |
| 7 | x | x | x | x | x |  | x |  |  |  | x | 172.80 |  |
| 8 | x | x | x | x | x |  | x |  | x |  | x | 174.62 |  |
| 9 | x | x | x | x | x |  | x | x | x |  | x | 176.48 |  |
| 10 | x | x | x | x | x |  | x | x | x | x | x | 178.40 |  |
| SWD FWDselect results. q = size of the considered covariate subset; x = covariate selected by the FWDselect algorithm at size q; AIC = Akaike information criterion; p-value = p-value regarding the hypothesis H1(q): the model fit improves including additional covariates. | | | | | | | | | | | | | |

Supplementary Results - Figure 1 | Heterogeneity of Regression Slopes


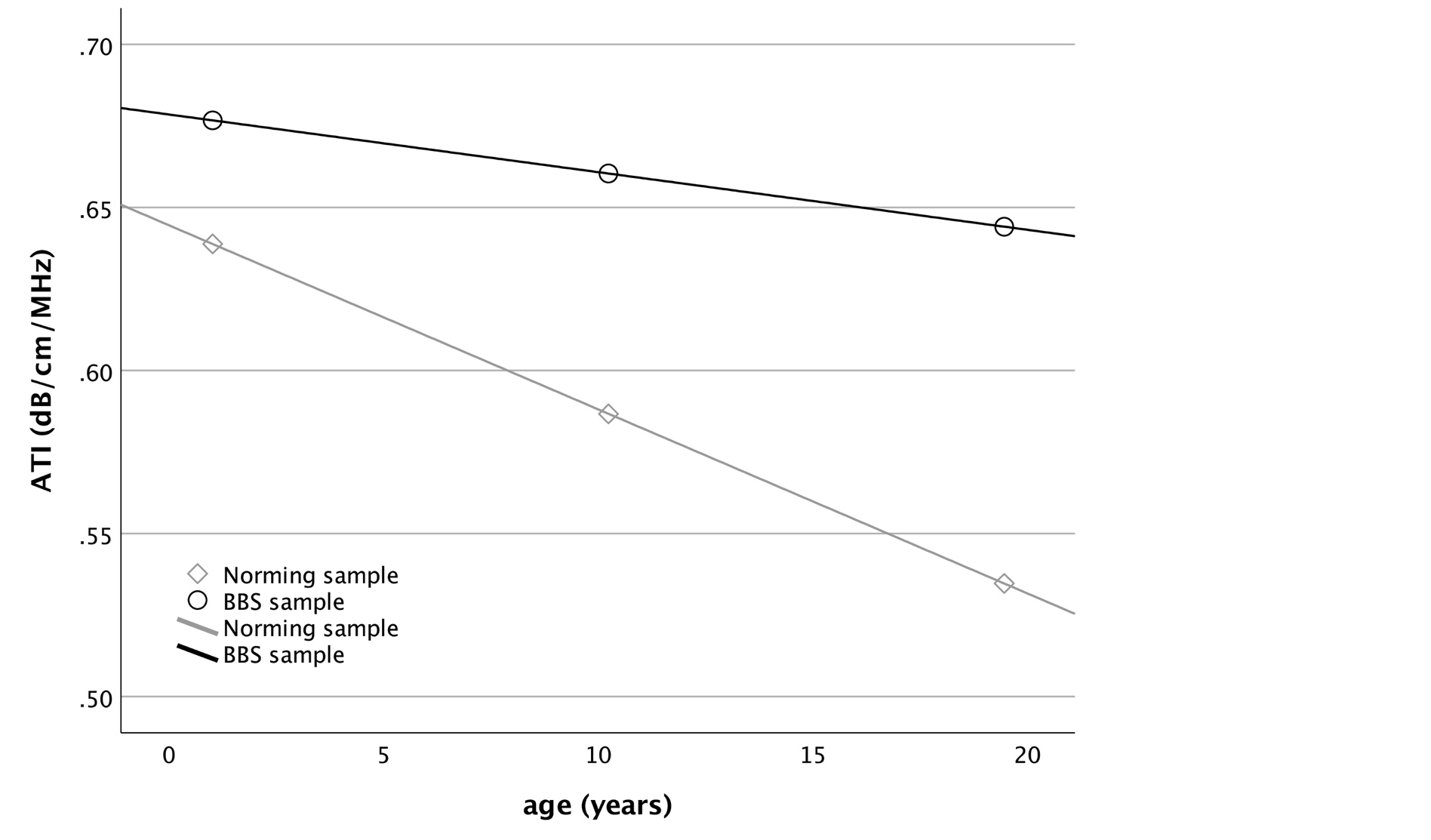


Relationship between ATI level (attenuation imaging coefficient; y-axis) in BBS patients versus the norming sample, with age (x-axis) analyzed at exemplary ages (-1 SD [14th percentile], the mean, and +1 SD [86th percentile]) as a moderator, and liver size (as a percentage), liver echogenicity, and BMI standard deviation scores (BMI-SDS) as covariates. Further details and the statistical significance of these relationships are discussed in the manuscript.
